# Empagliflozin and its impact on hepatic and metabolic outcomes in patients with type 2 diabetes and NAFLD: a systematic review and meta-analysis

**DOI:** 10.1186/s13098-025-02084-x

**Published:** 2026-01-08

**Authors:** Khadeeja Ali Hamzah, Mohammedsadeq A. Shweliya, Yousif Hameed Kurmasha, Marafi Jammaa Ahmed, Ashna Habib, Abanoub I.I. Kamel, Zarwa Rashid, Aya Ahmed Shimal, Mayar Moghazy, Fatima Fahem, Mohammad Yassin Al Aboud, Ahmed Elgazzar, Abdulhadi M. A. Mahgoub, Ali Saad Al-Shammari

**Affiliations:** 1 Department of Internal Medicine, Al-Kindy College of Medicine, University of Baghdad, Baghdad, Iraq; 2 Department of Internal Medicine, College of Medicine, University of Baghdad, Baghdad, Iraq; 3 Department of Internal Medicine, College of Medicine, University of Kufa, Najaf, Iraq; 4 Department of Internal Medicine, Faculty of Medicine, Bahri University, Khartoum, Sudan; 5 Department of Internal Medicine, Dow University of Health Sciences, Karachi, Pakistan; 6 Faculty of Pharmacy, Minia University, Minia, Egypt; 7 Department of Internal Medicine, King Edward Medical University, Lahore, Pakistan; 8 Department of Internal Medicine, Faculty of Medicine, Kafr El-Sheikh University, Kafr El-Sheikh, Egypt; 9 Department of Internal Medicine, College of Medicine, University of Al-Qadisiyah, Al- Qadisiyah, Iraq; 10 Department of Internal Medicine, Faculty of Medicine, Latakia University, Latakia, Syria; 11 Department of Internal Medicine, Faculty of Medicine, University of Gezira, North Sudan, Dongola, Suda Sudan; 12 Department of Cardiology, Camden Clark Medical Center, West Virginia University, Parkersburg, WV USA

**Keywords:** Empagliflozin, Type 2 diabetes mellitus, NonAlcoholic fatty liver disease, Liver fat, Liver fibrosis, Meta-analysis, SGLT2 inhibitors

## Abstract

**Background:**

Metabolic dysfunction–associated steatotic liver disease (MASLD), formerly nonalcoholic fatty liver disease (NAFLD), often coexists with type 2 diabetes mellitus (T2DM) due to shared metabolic pathways such as insulin resistance. Empagliflozin, a sodium-glucose cotransporter-2 (SGLT2) inhibitor, may provide hepatic and metabolic benefits. This study evaluated its effects on liver fat, enzymes, fibrosis, metabolic parameters, and inflammation in T2DM with MASLD.

**Methods:**

A systematic review and meta-analysis of randomized controlled trials (RCTs) was performed according to PRISMA guidelines. Primary outcomes included liver fat content, enzymes, and fibrosis markers. Secondary outcomes were metabolic and inflammatory parameters.

**Results:**

Eleven RCTs (*n* = 3077) were included. Empagliflozin significantly reduced liver fat (MD = -3.11%; 95% CI: -4.12 to -2.11; *p* < 0.00001) and liver stiffness (MD = -0.43 kPa; *p* = 0.003), but had no significant effect on AST (-0.27 IU/L; *p* = 0.89) or GGT (-9.25 IU/L; *p* = 0.14). It significantly lowered HbA1c (-0.54%; *p* < 0.0001), fasting glucose (-20.89 mg/dL; *p* < 0.0001), weight (-2.04 kg; *p* < 0.0001), and waist circumference (-3.47 cm; *p* < 0.0001), with a nonsignificant reduction in BMI (-0.77 kg/m²; *p* = 0.09).Uric acid decreased (-0.41 mg/dL; *p* < 0.00001), but IL-6 and fibrosis scores (FIB-4, NFS) remained unchanged.

**Conclusion:**

Empagliflozin improves liver fat, stiffness, glycemic control, body weight, and uric acid in T2DM with MASLD, but its effects on fibrosis and inflammation remain uncertain. Larger, long-term histologic trials are needed to confirm these outcomes.

## Introduction

Type 2 diabetes mellitus (T2DM) is a chronic, progressive metabolic disorder and a leading cause of morbidity and premature mortality worldwide, with approximately one death every 10 s [1]. Metabolic dysfunction-associated steatotic liver disease (MASLD), formerly referred to as nonalcoholic fatty liver disease (NAFLD), a metabolic liver disorder, is the most prevalent liver disease globally [2]. It encompasses a broad spectrum of liver damage, ranging from simple steatosis to nonalcoholic steatohepatitis (NASH), fibrosis, cirrhosis, and related complications. MASLD is strongly associated with T2DM and affects ~ 75% of T2DM patients [3]. Obesity and insulin resistance are key pathogenic factors of both MASLD and T2DM, explaining their frequent coexistence [4].

MASLD is characterized by excessive hepatic lipid accumulation and fibrosis in the absence of significant alcohol consumption [2]. In patients with T2DM, MASLD develops due to insulin resistance, which increases hepatic free fatty acid influx and de novo lipogenesis while impairing lipid oxidation. This lipid accumulation (‘first hit’) progresses to inflammation and fibrosis (‘second hit’) under oxidative stress, exacerbated by obesity and metabolic syndrome. MASLD and T2DM share a bidirectional relationship, where each condition worsens the other, further elevating cardiovascular risk [5].

Empagliflozin, a sodium-glucose cotransporter 2 (SGLT-2) inhibitor, is a novel oral hypoglycemic agent that inhibits renal glucose reabsorption, ameliorates insulin resistance, and downregulates SREBP-1c, thereby blocking de novo lipogenesis and improving lipid metabolism [6]. It also exerts anti-inflammatory and anti-fibrotic effects. It reduces markers of liver injury (AST/ALT) and may suppress pro-inflammatory cytokines (e.g., IL-6, TNF-α), thereby mitigating hepatic inflammation. It decreases liver stiffness (LSM), suggesting inhibition of fibrosis progression, potentially through improved metabolic parameters and reduced oxidative stress [7].

Empagliflozin’s mechanisms suggest therapeutic potential for MASLD in T2DM. These mechanisms are supported by preclinical studies and confirmed in high-quality RCTs, including Chehrehgosha et al. 2021, demonstrating that empagliflozin improves liver steatosis and fibrosis in patients with MASLD and T2DM effectively, reduces body weight and visceral fat area [8].

Despite these dual metabolic and hepatic benefits, high-quality evidence, Level 1 through meta-analysis, regarding empagliflozin’s effects on MASLD in T2DM patients remains limited. Due to the heterogeneous populations and outcomes across studies, this review aims to synthesize evidence on empagliflozin’s effects on: [1] hepatic outcomes: liver fat content (MRI-PDFF, MRI-proton density fat fraction/CAP, controlled attenuation parameter), enzyme levels (ALT, AST, GGT), and fibrosis scores (LSM, Liver Stiffness Measurement, FIB-4, Fibrosis-4 Index); [2] metabolic parameters (weight, HbA1c, and HOMA-IR); and [3] inflammatory markers (CRP and cytokines).

## Methods

**Fig. 1:**
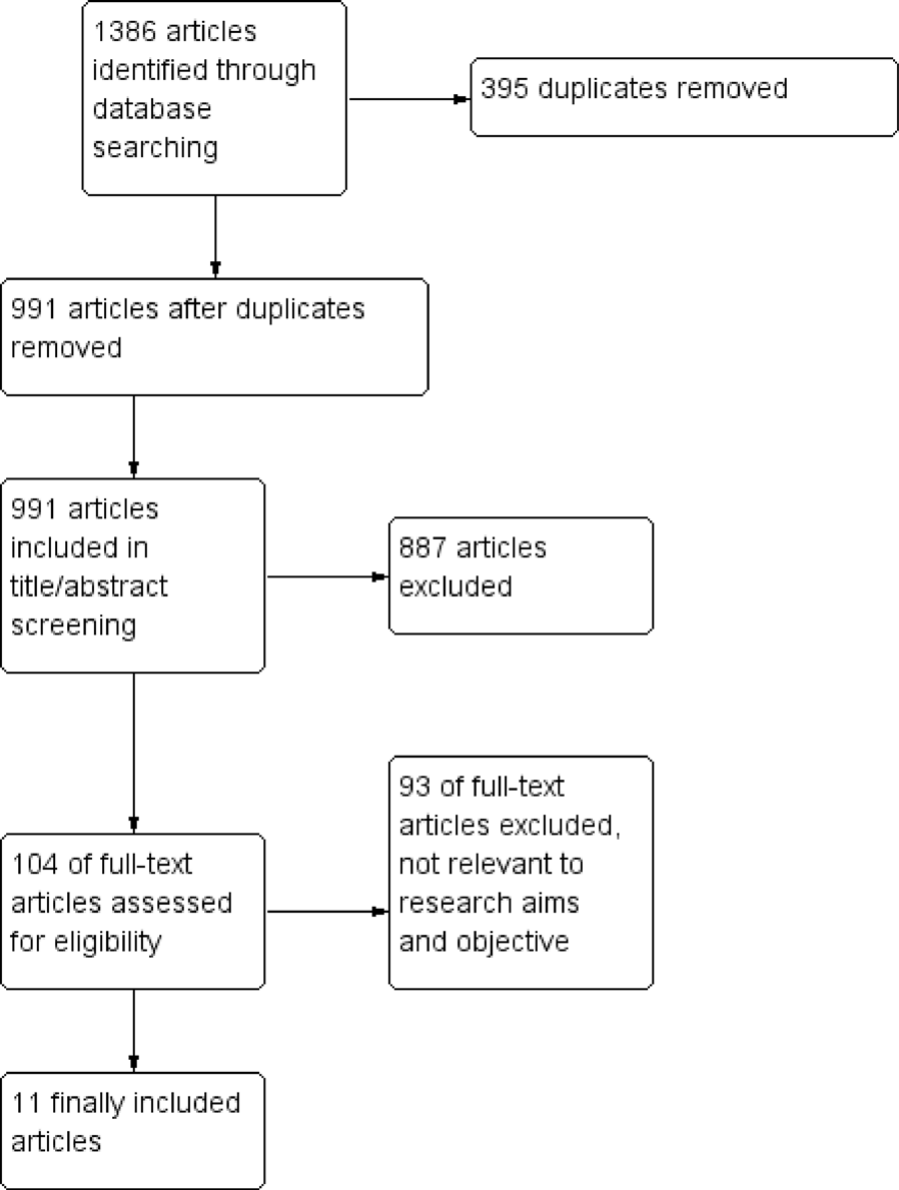
Prisma flow diagram

The present systematic review and meta-analysis was conducted in accordance with the Preferred Reporting Items for Systematic Reviews and Meta-Analyses (PRISMA) guidelines. We followed the PRISMA checklist to ensure methodological rigor. Figure 1 illustrates the search process and study selection. The study protocol was registered with PROSPERO (CRD420251077251) prior to data extraction.

### Data sources and search strategy

A literature search was conducted in Medline (through PubMed), Scopus, Embase, and Web of Science. The subject terms were as follows: “empagliflozin”, “NAFLD”, and “T2DM”. A broad search filter was applied to identify all studies, synonyms to the terms used were found on Medical Subject Headings (Mesh), and the terms were connected by ‘OR’ and ‘AND’ operators from the Cochrane Handbook for Systematic Reviews.

### Study selection and eligibility criteria

We included studies that met the following criteria: (a) Population: adult patients (≥ 18 years) diagnosed with type 2 diabetes mellitus (T2DM) and NAFLD or MASLD, Although most included studies used the NAFLD definition, we adopted the updated nomenclature of MASLD; acknowledging the considerable overlap between these entities. confirmed by: 1- Imaging (ultrasound, MRI-proton density fat fraction [MRI-PDFF] > 5%, or fibroscan-controlled attenuation parameter [CAP] ≥ 238 dB/m). 2- Liver biopsy (if applicable, e.g., steatosis with inflammation or ballooning). (b) Intervention: treatment with empagliflozin (any dose, as monotherapy or add-on therapy). (c) Comparator: placebo, standard care, or other glucose-lowering agents (e.g., metformin, sulfonylureas, GLP-1 receptor agonists, or other SGLT2 inhibitors). (d) Outcomes: changes in liver fat content assessed by imaging or biopsy and liver enzymes (ALT, AST) as the primary outcomes. Secondary outcomes included: [1] glycemic control (HbA1c, fasting glucose); [2] metabolic parameters (BMI, lipids, insulin resistance, uric acid); [3] liver fibrosis markers (LSM, NFS, FIB-4); and [4] inflammatory markers (e.g., IL-6). (e) Study Design: randomized controlled trials (RCTs), non-randomized controlled trials, or prospective/retrospective cohort studies. We excluded studies based on the following: (a) Population: non-T2DM patients (e.g., type 1 diabetes, gestational diabetes) or excessive alcohol consumption (> 20–30 g/day for men; >10–20 g/day for women). (b) Duplicate publications, (c) review articles, meta-analyses, case reports, conference abstracts, replies, letters to editors, book chapters, and comments. (d) Non-English language studies, as well as (e) animal and in vitro studies. Each publication that satisfied the selection criteria was evaluated independently by two authors; full texts of articles were screened in addition to the references list in each article to avoid missing any eligible studies. To resolve any conflicts, the data was reassessed by a different author who was not involved in the initial extraction process.

### Data extraction and outcomes

Three authors independently extracted relevant data for the meta-analysis into predesigned Excel spreadsheets. The extracted data included summary of the included studies (location, study arms, no. of patients (treatment/control), study population, intervention group, control group, dosage (intervention), dosage (control), primary efficacy outcome and follow up duration), baseline characteristics (demographic information such as age and sex distribution (number and percentage of males and females), anthropometric measurements including weight (kg), body mass index (BMI), and waist circumference, Diabetes-related parameters as diabetes duration (years), fasting glucose levels, and HbA1c values, Cardiovascular and metabolic markers included hypertension prevalence (number and percentage of patients), systolic and diastolic blood pressure measurements, Liver function tests (AST and ALT levels) and lipid profile components (triglycerides, LDL cholesterol, and HDL cholesterol), All continuous variables were recorded as mean ± standard deviation; when means are not reported, medians with interquartile ranges were extracted instead) and clinical outcomes. The outcomes included are changes in : [1] liver fat content assessed by imaging modalities (MRI-PDFF, CAP, or volume-selective proton MRS); [2] liver enzymes (AST, GGT); [3] glycemic parameters (HbA1c, fasting blood glucose); [4] lipid profile (triglycerides, LDL, HDL, total cholesterol); [5] anthropometric measures (weight, BMI, waist circumference); [6] blood pressure (systolic and diastolic); [7] liver fibrosis markers (LSM in kPa, NFS, FIB-4 index); and [8] additional metabolic and inflammatory markers (e.g., albumin, insulin sensitivity, uric acid, IL-6). For all outcomes, we extracted mean ± standard deviation values at baseline, post-treatment, and for the change from baseline.

### Quality assessment

Two independent authors assessed the quality of all included studies. The quality of RCTs was evaluated using the Cochrane Risk of Bias Assessment Tool, addressing the following specific domains: randomization process, deviations from the intended interventions, missing outcome data, measurement of the outcome and selective outcome reporting. Each domain was judged as “low risk”, “some concerns”, or “high risk” of bias, following the Cochrane Handbook guidelines. An overall risk of bias was assigned based on the highest risk level across domains. RevMan 5.4 was used for the quality assessment graph.

### Data synthesis and statistical analysis

All meta-analyses were performed using Review Manager 5.4, employing random-effects models (DerSimonian-Laird method) to account for anticipated clinical and methodological heterogeneity. For continuous outcomes, pooled estimates were calculated as mean differences (MD) with 95% confidence intervals (CIs). Categorical variables were analyzed using relative risks (RRs) with 95% CIs. P values less than 0.05 were considered statistically significant. Heterogeneity was assessed using the: [1] I² statistic, interpreted as follows: 25%: Low heterogeneity, 50%: Moderate heterogeneity and 75%: High heterogeneity [2]. Cochran’s Q test (χ²), where a p-value < 0.10 indicated significant heterogeneity. To solve the heterogeneity and to evaluate the stability of our results, we performed leave-one-out sensitivity analyses to assess whether pooled estimates were driven by individual outlier studies, examining changes in effect size, p-values, and I². Publication bias was assessed visually via funnel plots using RevMan 5.4.

## Results

### Search results

A total of 1,386 articles were identified through systematic database searching. After removing 395 duplicates, 991 articles remained for screening. Title and abstract screening led to the exclusion of 887 articles that did not meet the inclusion criteria. Subsequently, 104 full-text articles were assessed for eligibility. Of these, 93 were excluded as they were not relevant to the research aims and objectives. Finally, 11 studies were included in the final analysis. The included studies were selected based on their relevance to the predefined eligibility criteria, and the full selection process is illustrated in the PRISMA flow diagram (Fig. 1).

### Characteristics and quality assessment

Eleven randomized controlled trials (RCTs) conducted between 2018 and 2025 were included [9–19], including a total of 3077 participants. Across all studies, 1977 participants received empagliflozin-based therapies, while 1100 were assigned to control groups (placebo, standard-of-care, or metformin monotherapy). The studies were carried out in diverse geographical locations, including Greece, India, Italy, Japan, and multiple international centers. All studies consisted of a parallel group RCT design and included adults diagnosed with type 2 diabetes mellitus (T2DM) who had confirmed MASLD. Empagliflozin was typically administered at 10 mg daily, either as monotherapy or in combination with standard antidiabetic regimens. Follow-up durations ranged from 12 weeks to a median of 3.1 years. The primary efficacy outcomes focused on hepatic fat reduction and liver stiffness, assessed via MRI-PDFF, CAP, or elastography. Full study details are provided in the (Table 1). A summary of the included studies is shown in (Table 2).

**Table 1:** Baseline characteristics

	Study groups	Age, years	Sample size, male/female	male %	female %	Weight, Kg	BMI, (kg/m²)	Waist CircumFerence (cm)	Durati on of Diabetes, years	Fasting Glucose ,mg/dl	HbA1c,%	SBP (mmHg)	DBP (mmHg)	AST (IU/L)	ALT (IU/L)	TG (mg/dL)	LDL (mg/dL)	HDL (mg/dL)
Koullias et al. 2024	Empagliflozin	63.24 ± 1.45	25 (18/7)	18 (72%)	7 (28%)	85.16 ± 3.56	29.64 ± 0.91	Men: 103.8 ± 3.1 & Women: 107.4 ± 3.1	NA	131.5 ± 9.3	7.23 ± 0.21	NA	NA	30.1 ± 3.2	39.9 ± 4.0	134.8 ± 10.8	74.4 ± 6.1	40.7 ± 1.5
	control	55.00 ± 2.38	28 (17/11)	17 (60.7%)	11 (39.3%)	93.41 ± 3.75	31.53 ± 1.00	Men: 112.8 ± 3.4 & Women: 98.5 ± 8.3	NA	110.5 ± 4.5	6.40 ± 0.14	NA	NA	27.1 ± 2.4	40.1 ± 6.7	129.6 ± 11.5	101.9 ± 7.1	43.5 ± 1.9
Kuchay et al. 2018	Empagliflozin	Not reported	Not reported	Not reported	Not reported	80.8 ± 13.0	30.0 ± 3.8	NA	NA	173 ± 44	9.0 ± 1.0	125 ± 13	79 ± 10	44.6 ± 23.5	64.3 ± 20.2	201 ± 124	112 ± 35	42 ± 12
	control	Not reported	Not reported	Not reported	Not reported	81.1 ± 16.1	29.4 ± 3.1	NA	NA	176 ± 57	9.1 ± 1.4	130 ± 19	81 ± 12	45.3 ± 24.3	65.3 ± 40.3	212 ± 115	114 ± 30	45 ± 15
Caturano et al. 2023	Empagliflozin	61.90 ± 10.88	17/13	56.70%	43.30%	NA	30.83 ± 3.52	NA	NA	NA	8.20 [7.40, 8.80]	NA	NA	44.00 [28.50, 47.75]	68.5 [41.5, 88.0]	NA	NA	NA
	control	60.09 ± 11.47	19/14	57.60%	42.40%	NA	31.89 ± 4.65	NA	NA	NA	7.10 [6.50, 7.50]	NA	NA	38.00 [26.00, 42.00]	49.00 [32.00, 67.00]			
Esmaeili et al. 2024	Empagliflozin	53.26 ± 7.64	18/12	60%	40%	NA	NA	NA	NA	NA	NA	NA	NA	NA	NA	NA	NA	NA
	control	Not Reported	17/13	56.70%	43.30%	NA	NA	NA	NA	NA	NA	NA	NA	NA	NA	NA	NA	NA
Sakurai et al. 2020	Empagliflozin	58.6 ± 12.9	16/15	51.60%	48.40%	70.0 ± 19.1	27.2 ± 5.7	97.6 ± 12.7	NA	173.0 ± 49.4	8.00 ± 0.86	134.5 ± 19.5	76.9 ± 12.0	22.5 (17, 30.5)	27.5 (13, 44.8)	122.2 ± 67.9	93.3 ± 24.5	54.4 ± 16.3
	control	58.6 ± 12.2	12/6	66.70%	33.30%	74.4 ± 18.6	28.4 ± 6.6	100.9 ± 11.5	NA	146.3 ± 33.8	7.63 ± 0.55	140.9 ± 21.5	75.2 ± 11.0	25 (15.8, 38.5)	22 (14, 34.3)	112.8 ± 57.7	100.6 ± 28.2	54.2 ± 15.0
Kahl et al. 2020	Empagliflozin	62.7 ± 7.0	29/13	69%	31%	NA	32.1 ± 4.6	NA	36 ± 27	7.5 ± 1.4	6.8 ± 0.5	NA	NA	0.42 (0.36–0.49)	0.54 (0.42–0.80)	159 (122–202)	133 ± 40	50 ± 15
	control	61.5 ± 10.0	29/13	69%	31%	NA	32.4 ± 4.2	NA	40 ± 27	7.5 ± 1.4	6.7 ± 0.7	NA	NA	0.43 (0.37–0.55) |	0.62 (0.42–0.88)	181 (103–251)	120 ± 30	48 ± 10
Chehrehgosha et al. 2021	Empagliflozin	51.8 ± 7.8	14/23	14 (37.8%)	23 (62.2%)	80.3 ± 12.8	30.2 ± 4.4	NA	7.0 ± 5.3	156.3 ± 34.0	7.96 ± 0.62	NA	NA	24.2 ± 10.7	32.1 ± 17.3	183.5 ± 127.7	91.3 ± 24.1	47.6 ± 9.4
	control	52.5 ± 7.9	17/18	17 (48.6%)	18 (51.4%)	82.2 ± 12.3	30.9 ± 3.3	NA	5.5 ± 4.0	149.6 ± 42.2	8.08 ± 0.92	NA	NA	26.0 ± 16.5	31.1 ± 16.9	183.2 ± 149.3	85.0 ± 15.1	45.6 ± 9.1
Jojima et al. 2021	Empagliflozin	58.6 ± 12.2	18 (12/6)	12 (66.7%)	6 (33.3%)	74.4 ± 18.6	28.4 ± 6.6	100.9 ± 11.5	NA	146.3 ± 33.8	7.6 ± 0.6	140.9 ± 21.5	75.2 ± 11.0	Not reported	Median 22 (IQR 14–343.3)	Mean not given; Median 112.8 (IQR 57.7)	100.6 ± 28.2	54.2 ± 15.0
	control	58.6 ± 12.9	31 (16/15)	16 (51.6%)	15 (48.4%)	70.0 ± 19.1	27.2 ± 5.7	97.6 ± 12.7	NA	173.0 ± 49.4	8.0 ± 0.9	134.5 ± 19.5	76.9 ± 12.0	NA	Median 27.5 (IQR 13–44.8)	Mean not given; Median 122.2 (IQR 67.9)	93.3 ± 24.5	54.4 ± 16.3
Sattar et al. 2018	Empagliflozin	NA	NA	71%	29%	86.3+−18.9	30.6+−5.3	NA	NA	NA	8.1+−0.85%	NA	NA	22.9+−10.3	26.2+−15.3	NA	NA	NA
	control	NA	NA	71%	29%	86.3+−18.9	30.6+−5.3	NA	NA	NA	8.1+−0.85%	NA	NA	22.5+−9.6	25.5+−13.8	NA	NA	NA
Abdelgani et al. 2024 (DM)	Empagliflozin	54 ± 2	20	12	8	−1.79 ± 0.25 kg	32.4 ± 1.1	NR	5.9 ± 1.5 years	149 ± 8 mg/dL	7.5 ± 0.4%	NA	NA	24 ± 2 IU/L	36 ± 4 IU/L	NA	NA	NA
	control	57 ± 2	10	5	5	NA	33.2 ± 1.2	NR	6.0 ± 2.1 years	159 ± 14 mg/dL	7.8 ± 0.5%	NA	NA	19 ± 1 IU/L	28 ± 3 IU/L	NA	NA	NA
Shojaei et al. 2025	Empagliflozin	46.32 ± 8.11	69 (38/31)	38 (54.29%)	31 (45.71%)	NA	32.18 ± 4.24	NA	NA	123.78 ± 26.52 mg/dL	6.59 ± 0.72%	118.19 ± 11.42 mmHg	73.55 ± 10.58 mmHg	46.74 ± 14.14 U/L	71.33 ± 49.35 U/L	NA	NA	NA
	control	52.56 ± 10.26	50 (32/18)	32 (64%)	18 (36%)	NA	31.13 ± 6.05	NA	NA	113.06 ± 13.15 mg/dL	6.63 ± 0.44%	121.10 ± 10.11 mmHg	74.50 ± 9.05	42.54 ± 11.61 U/L	47.52 ± 7.46 U/L	NA	NA	NA

**Table 2:** Summary of the included studies

Study id	Location	Study design	Study arms	No. of patients (Treatment/Control)	Study population	Intervention group	Control group	Dosage (Intervention)	Dosage (Control)	Primary efficacy outcome	Follow up duration
** Koullias et al. 2024**	Two Diabetes Centers in Athens, Greece.		Three Arms: • Empagliflozin group &• Dulaglutide group & • Control group	Total enrolled: 78 patients • Empagliflozin group: 25 patients • Dulaglutide group: 25 patients • Control group: 28 patients	Adults diagnosed with Type 2 Diabetes Mellitus (T2DM) and nonalcoholic fatty liver disease (NAFLD)	Patients receiving Empagliflozin added to their standard-of-care treatment	Patients receiving only standard-of-care treatment without the addition of Empagliflozin or Dulaglutide	Empagliflozin: 10 mg orally once daily	-N/A-	Reduction in liver fat fraction (LFF), assessed using magnetic resonance imaging-proton density fat fraction (MRI-PDFF)	52 weeks (1 year)
** Kuchay et al. 2018**	Gurugram, Haryana, India (conducted at Medanta-The Medicity Hospital)		Two Arms: •Empagliflozin group & • Control group	Total enrolled: 50 patients • Empagliflozin group: 25 patients • Control group: 25 patients	Adults diagnosed with Type 2 Diabetes Mellitus (T2DM) and nonalcoholic fatty liver disease (NAFLD)	Patients receiving Empagliflozin added to their standard-of-care treatment	Patients receiving only standard-of-care treatment without the addition of Empagliflozin	Empagliflozin: 10 mg orally once daily	-N/A-	Reduction in liver fat content, assessed using magnetic resonance imaging-derived proton density fat fraction (MRI-PDFF)	20 weeks
** Caturano et al. 2023**	Naples, Italy (conducted at the University of Campania Luigi Vanvitelli)		Two Arms: • Combination therapy group (Empagliflozin + Metformin) & • Monotherapy group (Metformin alone)	Total enrolled: 63 patients • Combination therapy group: 33 patients • Monotherapy group: 30 patients	Adults diagnosed with Type 2 Diabetes Mellitus (T2DM) and nonalcoholic fatty liver disease (NAFLD)	Patients receiving Empagliflozin added to their existing Metformin therapy	Patients receiving Metformin monotherapy without the addition of Empagliflozin	Empagliflozin: 10 mg orally once daily + Metformin: continued at pre-study dosage	Metformin only: continued at pre-study dosage	Change in liver stiffness measurement (LSM) assessed by transient elastography (FibroScan) to evaluate liver fibrosis progression	6 months
**Kahl et al. 2022**	Multinational (conducted across various countries)		Two Arms: • Empagliflozin group & • Placebo group	Total enrolled: 7,020 patients • Empagliflozin group: 4,687 patients • Placebo group: 2,333 patients	Adults with Type 2 Diabetes Mellitus (T2DM) and established cardiovascular disease	Patients receiving Empagliflozin in addition to standard-of-care treatment	Patients receiving placebo in addition to standard-of-care treatment	Empagliflozin: 10 mg or 25 mg orally once daily	-N/A-	Reduction steatosis but not fibrosis risk in individuals with type 2 diabetes and cardiovascular disease	Median of 3.1 years
** Esmaeili et al. 2024**	Japan (conducted at Single center)		Two Arms: • Empagliflozin add-on therapy group & • Metformin monotherapy group	Total enrolled: 60 patients • Empagliflozin + MET group: 30 patients • Metformin only group: 30 patients	Adults diagnosed with Type 2 Diabetes Mellitus (T2DM) and nonalcoholic fatty liver disease (NAFLD)	Patients receiving Empagliflozin added to their existing Metformin therapy	Patients receiving Metformin monotherapy without the addition of Empagliflozin	Empagliflozin: 10 mg orally once daily Metformin: 1500-2500 mg	Metformin: continued at 1500-2500 mg	Change in hepatic fat content assessed by magnetic resonance imaging-proton density fat fraction (MRI-PDFF)	24 weeks
** Sakurai et al. 2020**	Japan (Dokkyo Medical University)		Two Arms: • Empagliflozin group & • Control group	Total enrolled: 49 patients • Empagliflozin group: 31 patients • Control group: 18 patients	Adults diagnosed with Type 2 Diabetes Mellitus (T2DM)	Patients receiving Empagliflozin in addition to standard therapy	Patients receiving standard therapy without the addition of Empagliflozin	Empagliflozin: 10 mg orally once daily	-N/A-	Change in plasma concentration of plasminogen activator inhibitor-1 (PAI-1) as an indicator of fibrinolytic activity	12 weeks
**Kahl et al. 2020**	Conducted at five centers in Germany: Düsseldorf, Potsdam-Rehbrücke, Dresden, Tübingen, and Heidelberg		• Empagliflozin 25 mg/day • Placebo	• Total randomized: 84 • Empagliflozin group: 42 • Placebo group: 42	• Adults aged 18–75 years with well-controlled, recent-onset type 2 diabetes • Mean diabetes duration: approx. 3 years • HbA1c: 6–8% • BMI < 45 kg/m² • No prior antihyperglycemic therapy or post-washout	Empagliflozin 25 mg orally once daily	Placebo tablet (matching in appearance)	25 mg/day empagliflozin	Placebo, once daily, matched in appearance and dosing	Change in liver fat content (LFC) measured by magnetic resonance spectroscopy after 24 weeks	24 weeks
**Lai et al. 2019**	University of Malaya Medical Centre, Kuala Lumpur, Malaysia		Single-arm, open-label, pilot study	• Treatment: 9 patients received empagliflozin • Control: Historical placebo group from a previous study (no concurrent control group)	Adults with type 2 diabetes mellitus and biopsy-proven nonalcoholic steatohepatitis (NASH)	Patients received empagliflozin 25 mg daily for 24 weeks	Historical placebo group from a previous 48-week clinical trial at the same center	Empagliflozin 25 mg daily	Placebo (from prior trial)	Changes in histological components of NASH (steatosis grade, lobular inflammation grade, hepatocyte ballooning grade, and fibrosis stage); NASH resolution without worsening fibrosis	
** Chehrehgosha et al. 2021**	Iran University of Medical Sciences (IUMS), Tehran, Iran		Three groups – Empagliflozin, Pioglitazone, and Placebo	• Empagliflozin: 35 • Pioglitazone: 34 • Placebo (Control): 37	Adults (20–65 years old) with Type 2 Diabetes Mellitus (T2DM) and NonAlcoholic Fatty Liver Disease (NAFLD	Empagliflozin 10 mg daily	Placebo	Empagliflozin 10 mg once daily	Placebo once daily	Change in liver steatosis measured by the Controlled Attenuation Parameter (CAP) score from baseline to 24 weeks	24 weeks
** Jojima et al. 2021**	Dokkyo Medical University, Tochigi, Japan.		• Intervention: Empagliflozin 10 mg/day. • Control: Standard therapy (no SGLT2 inhibitors).	• Treatment group: 32 (31 analyzed). • Control group: 19 (18 analyzed).	Adults with type 2 diabetes; excluded if they had type 1 diabetes, renal insufficiency (eGFR < 45), liver dysfunction, or used ezetimibe.	Empagliflozin 10 mg/day for 12 weeks.	Standard anti-diabetic therapy (metformin, DPP-4 inhibitors, sulfonylureas, insulin), no SGLT2 inhibitors.	Empagliflozin 10 mg once daily.	Not standardized; standard care without SGLT2 inhibitors.	Change in plasma levels of campesterol and HDL cholesterol. (Plasma campesterol and HDL significantly increased in the empagliflozin group.)	12 weeks.
** Sattar et al. 2018**	• Multinational, with authors and study coordination from the UK (University of Glasgow), Canada (University of Toronto), and Germany (Boehringer Ingelheim).		• Empagliflozin 10 mg • Empagliflozin 25 mg • Placebo • Glimepiride (in head-to-head trial)	• EMPA-REG OUTCOME trial: • Placebo group: 2333 patients • Empagliflozin group (combined 10 mg & 25 mg): 4687 patients • Pooled 24-week trials: • Placebo: 825 • Empagliflozin: 1652 • EMPA-REG H2H-SU trial: • Empagliflozin: 765 • Glimepiride: 780	• Adults with type 2 diabetes, varying baseline HbA1c (7–10%), BMI ≤ 45 kg/m², and with or without established cardiovascular disease.	• Empagliflozin 10 mg or 25 mg, once daily	• Placebo or glimepiride 1–4 mg (in H2H trial)	• Empagliflozin 10 mg or 25 mg once daily	Placebo (0 mg, inactive comparator) • Glimepiride 1–4 mg/day, titrated based on fasting glucose levels	• Change in ALT (alanine aminotransferase) and AST (aspartate aminotransferase) levels (as correlates of liver fat); • For EMPA-REG OUTCOME: primary outcome was 3-point major adverse cardiovascular events; liver enzyme analysis was a secondary endpoint.	• EMPA-REG OUTCOME: up to 164 weeks • Pooled placebo-controlled trials: 24 weeks • EMPA-REG H2H-SU trial: 104 weeks
** Abdelgani et al. 2024**	University of Texas Health Science Center, San Antonio, Texas, USA.		• Empagliflozin 25 mg • Matching placebo	• With T2D: Empagliflozin *n* = 20, Placebo *n* = 10 • Without diabetes: Empagliflozin *n* = 18, Placebo *n* = 9 • Total: Empagliflozin *n* = 38, Placebo *n* = 19	Obese adults with or without type 2 diabetes, undergoing metabolic testing and MRI-based liver fat quantification.	Empagliflozin 25 mg orally once daily for 12 weeks.	Matching placebo once daily for 12 weeks	25 mg empagliflozin once daily.	Matching placebo once daily.	Change in liver fat content (absolute reduction measured by MRI). • Empagliflozin reduced liver fat by 2.39% ± 0.79% vs. increase of 0.91% ± 0.64% with placebo (*P* < 0.007)	12 weeks
**Shinozaki et al. 2020**	Japan (Tochigi/Kyoto)		empagliflozin (single arm)	24 (no control)	Adults with T2DM and NAFLD (BMI ≥ 24, HbA1c > 7%)	empagliflozin	none	10 mg/day	N/A	Improvement of markers of hepatic inflammation, function and f ibrosis during one-year empagliflozin treatment	one year
** Lin et al. 2024**	SHOULD BE EXCLUDED ONGOING STUDY WITH NO DATA										
**Pokharel et al. 2021**	Kathmandu, Nepal		empagliflozin (single arm)	84 (no control)	Adults with T2DM and NAFLD (BMI ≥ 24, HbA1c > 7%)	empagliflozin	none	10 mg/day	N/A	Liver fat reduction and increase in fibrosis regression	6 months
** Shojaei et al. 2025**	Laghman Hakim Hospital, Tehran, Iran.		two arms: empagliflozin vs. control	129 (69/50)	Patients with T2DM and NAFLD	empagliflozin	routine clinical management for T2DM & NAFLD	N/A	N/A	Reduction in liver fat grade (MRI/ultrasound)	6 months
**Amin et al. 2025**	Kasr Al-Aini Hospital, Cairo University, Egypt		empagliflozin (single arm)	30 (no control)	Patients with T2DM and MASLD	empagliflozin 10 mg/day and standard care of treatment	none	10 mg/day	N/A	Percentage change in hepatic fat mass (MRI-PDFF measurement), showing a 13.16% reduction in hepatic steatosis after 24 weeks	24 weeks

### Quality assessment

Risk of bias assessment for the included RCTs using the Cochrane risk of bias tool (ROB1) showed low risk regarding selection bias, detection bias, attrition bias, and reporting bias. However, Jojima et al. (2021) conducted an open-label study design, with only blinding for the outcome assessment (Table 3).

Regarding non-randomized trials, studies assessed to be of good quality using the Newcastle–Ottawa Scale (NOS) (Table 4).

**Table 3:** ROB-2 evaluation of RCTs

Study ID	D1 (Risk of bias from the randomization process)	D2 (Risk of bias due to deviations from intended interventions)	D3 (Risk of bias due to missing outcome data)	D4 (Risk of bias in measurement of the outcome)	D5 (Risk of bias in selection of the reported result)	D6 (Overall Risk of bias)
Kuchay 2018	low	some concerns	low	low	low	some concerns
Sakurai 2020	low	some concerns	low	low	low	some concerns
Chehrehgosha 2021	low	low	low	low	low	low
Jojima 2021	low	some concerns	low	low	some concerns	some concerns
Sattar 2018	sub study	low	low	low	some concerns	some concerns
Shojaei 2025	some concerns	some concerns	some concerns	low	some concerns	some concerns
Abdelgani 2024	some concerns	low	some concerns	low	low	some concerns
Kahl 2020	low	low	low	low	low	low

**Table 4:** NOS quality assessment for cohort studies

	Selection				Comparability	Outcome			
ID	Representativeness of the exposed cohort	Selection of the non exposed cohort	Ascertainment of exposure	Demonstration that outcome of interest was not present at start of study	Comparability of cohorts on the basis of the design or analysis	Assessment of outcome	Was follow-up long enough for outcomes to occur	Adequacy of follow up of cohorts	Quality Score
Caturano, 2023	*	*	*	*	**	*	*	*	9
Koullias 2024	*	*	*	*	*	*	*	*	8
Esmaeili 2024	*	*	*	*	*	*	*	*	8

### Meta-analysis

#### Liver fat content

**Fig. 2:**
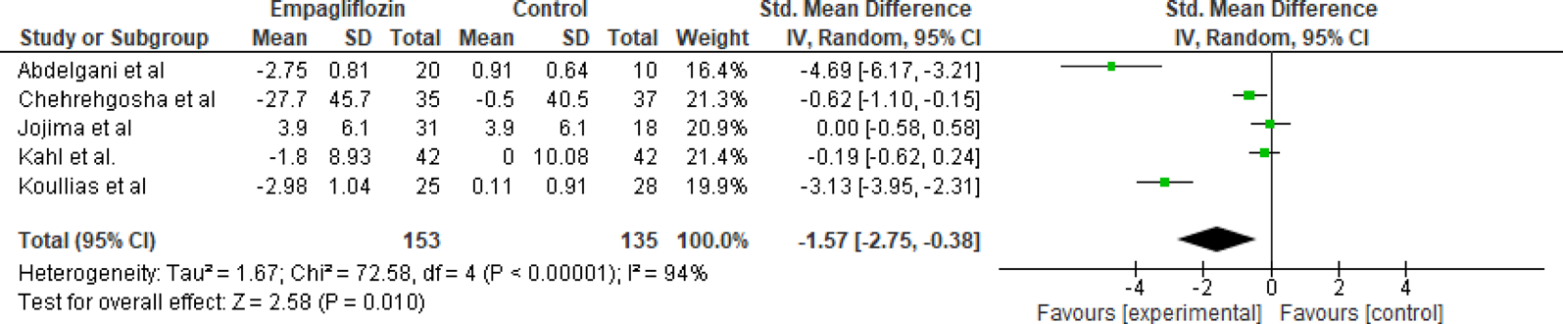
Forest plot of empagliflozin vs. control on liver fat content; Empagliflozin significantly reduced liver fat content compared with control (Std. MD = −1.57; 95% CI: −2.75 to −0.38; *p* < 0.001)

Five studies assessed the impact of empagliflozin versus standard care on liver fat content, evaluated using imaging modalities such as MRI-PDFF, CAP, and volume-selective proton MRS, with a total of 153 participants in the empagliflozin group and 135 participants in the control group. The pooled analysis demonstrated a statistically significant reduction in liver fat content in the empagliflozin group compared to the control group (Std. MD = −1.57; 95% CI: −2.75 to −0.38; *p* < 0.001). High heterogeneity was observed among the included studies (Tau² = 1.67; Chi² = 72.58, df = 4; *p* = 0.02; I² = 94%). The reduction suggests a meaningful improvement in hepatic steatosis despite study variability (Fig. 2).

#### AST

**Fig. 3:**
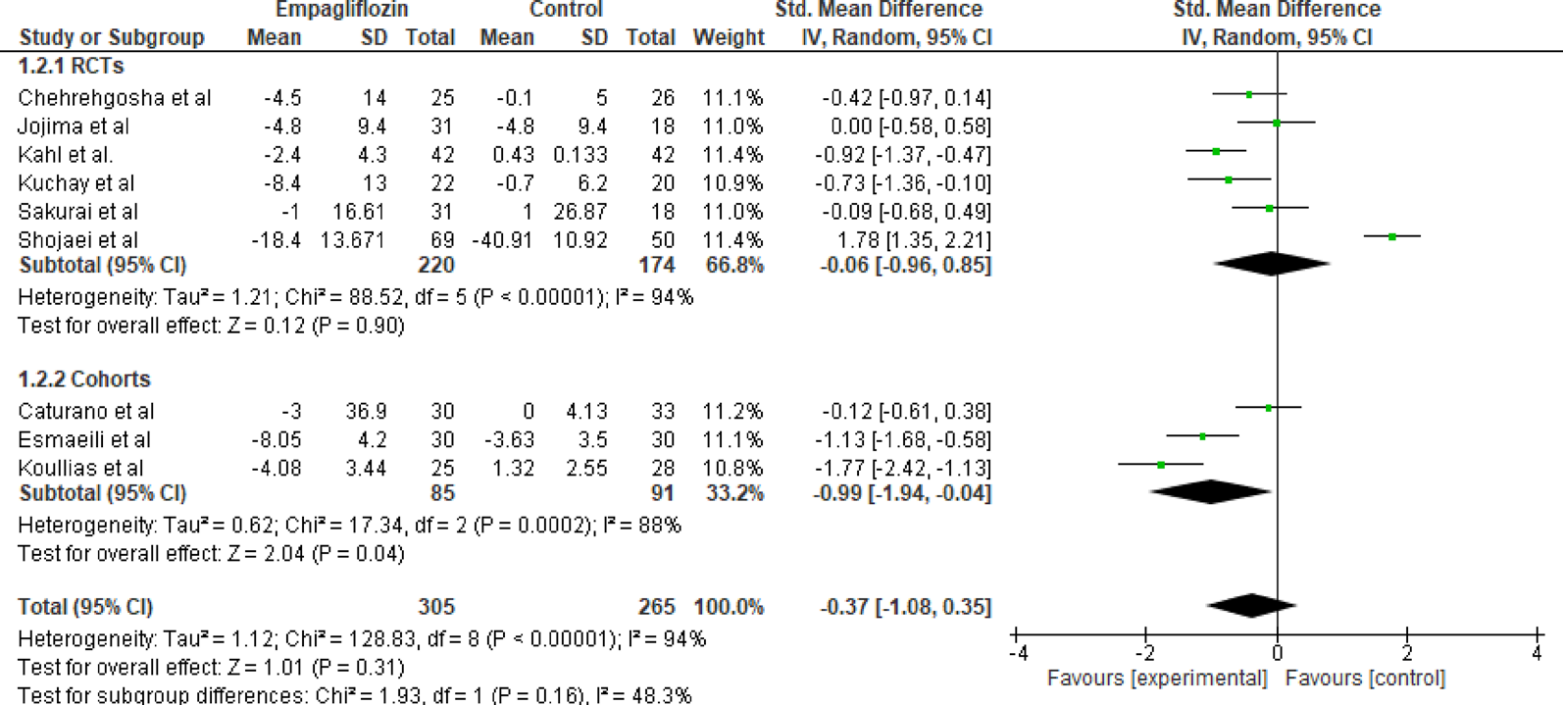
Forest plot of empagliflozin vs. control on AST levels; Empagliflozin did not significantly alter AST levels compared with control (SMD = −0.37; 95% CI: −1.08 to 0.35; *p* = 0.31)

Nine studies evaluated the effect of empagliflozin on AST levels in patients with type 2 diabetes mellitus. In the RCT subgroup, no significant difference in AST change was observed between empagliflozin and control groups (SMD = −0.06, 95% CI: −0.96 to 0.85; *p* = 0.90), with considerable heterogeneity (I² = 94%, Tau² = 1.21, Chi² = 88.52, df = 5, *p* < 0.00001). In the cohort studies subgroup, a reduction in AST was noted (SMD = −0.99, 95% CI: −1.94 to −0.04; *p* = 0.04), though results were highly heterogeneous(I² = 88%, Tau² = 0.62, Chi² = 17.34, df = 2, *p* = 0.0002). The overall pooled analysis from all studies showed no significant change in AST between groups (SMD = −0.37, 95% CI: −1.08 to 0.35; *p* = 0.31), with high heterogeneity (I² = 94%, Tau² = 1.12, Chi² = 128.83, df = 8, *p* < 0.00001). No significant subgroup difference was detected (*p* = 0.16). The wide CI indicates uncertainty rather than absence of effect; mild enzyme changes may still be relevant (Fig. 3).

#### GGT

**Fig. 4:**
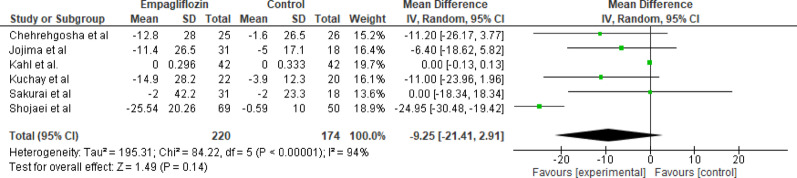
Forest plot of empagliflozin vs. control on GGT levels; Empagliflozin showed no significant change (MD = −9.25; 95% CI: −21.41 to 2.91; *p* = 0.14)

Six studies evaluated the effect of empagliflozin versus standard care on serum GGT levels, with a total of 220 participants in the empagliflozin group and 174 participants in the control group. The pooled analysis showed a nonsignificant trend toward lower GGT levels; however, the results were highly variable across studies. No significant change was observed (MD = −9.25; 95% CI: −21.41 to 2.91; *p* = 0.14). Substantial heterogeneity was detected among the studies (Tau² = 195.31; Chi² = 84.22, df = 5; *p* < 0.00001; I^2^ = 94%), suggesting variability in study results and possible inconsistent hepatic response (Fig. 4).

#### HbA1c

**Fig. 5:**
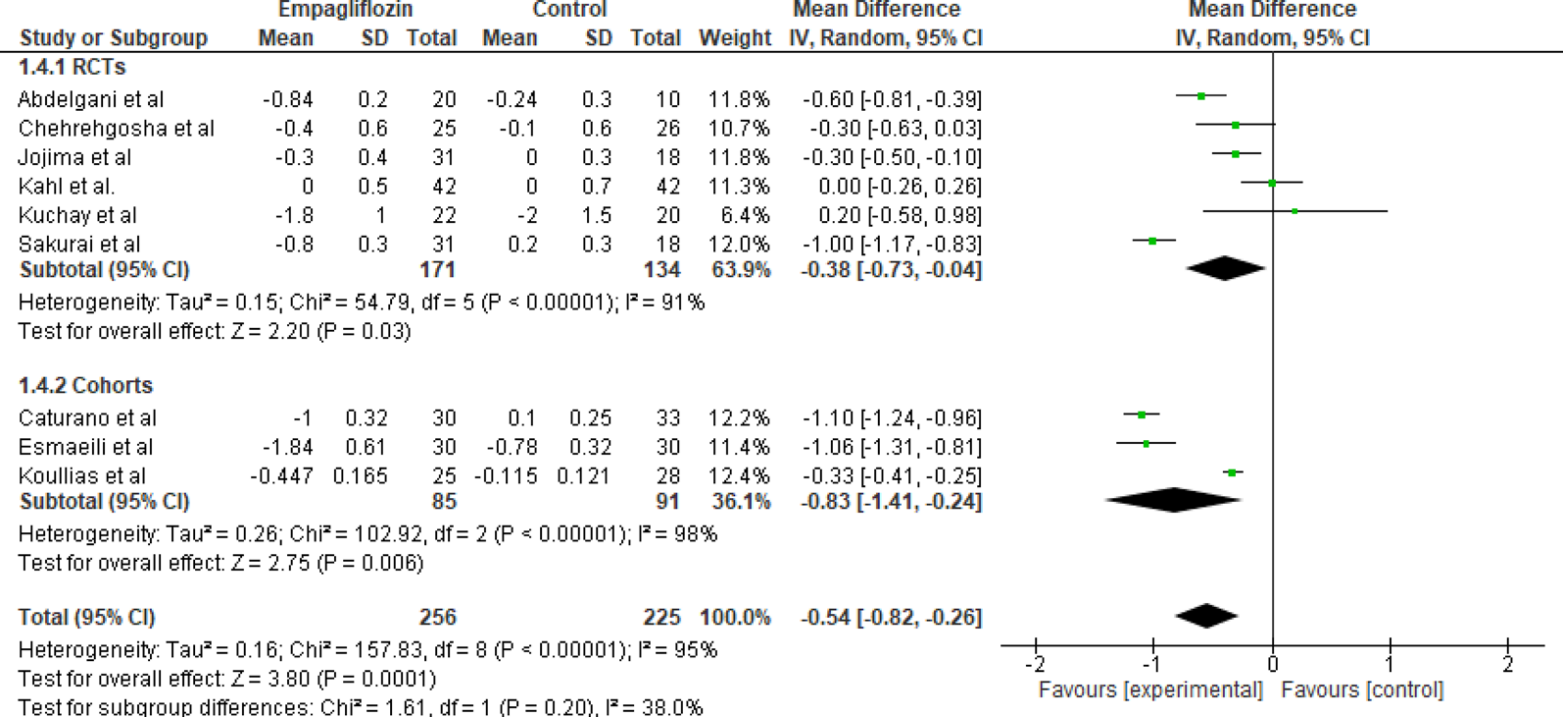
Forest plot of empagliflozin vs. control on HbA1c. Empagliflozin significantly reduced HbA1c compared with control (MD = −0.54%; 95% CI: −0.82 to −0.26; *p* < 0.001)

Nine studies evaluated the effect of empagliflozin versus standard care on HbA1c levels with a total of 256 participants in the empagliflozin group and 225 participants in the control group. The pooled analysis showed a statistically significant reduction in HbA1c levels with empagliflozin (MD = −0.54%; 95% CI: −0.82 to −0.26; *p* < 0.001). Substantial heterogeneity was detected among the studies (Tau² = 0.16; Chi² = 157.83, df = 5; *p* < 0.001; I^2^ = 95%). The decrease is both statistically and clinically meaningful, confirming glycemic benefit (Fig. 5).

#### Fasting blood glucose (FBS) levels

**Fig. 6:**
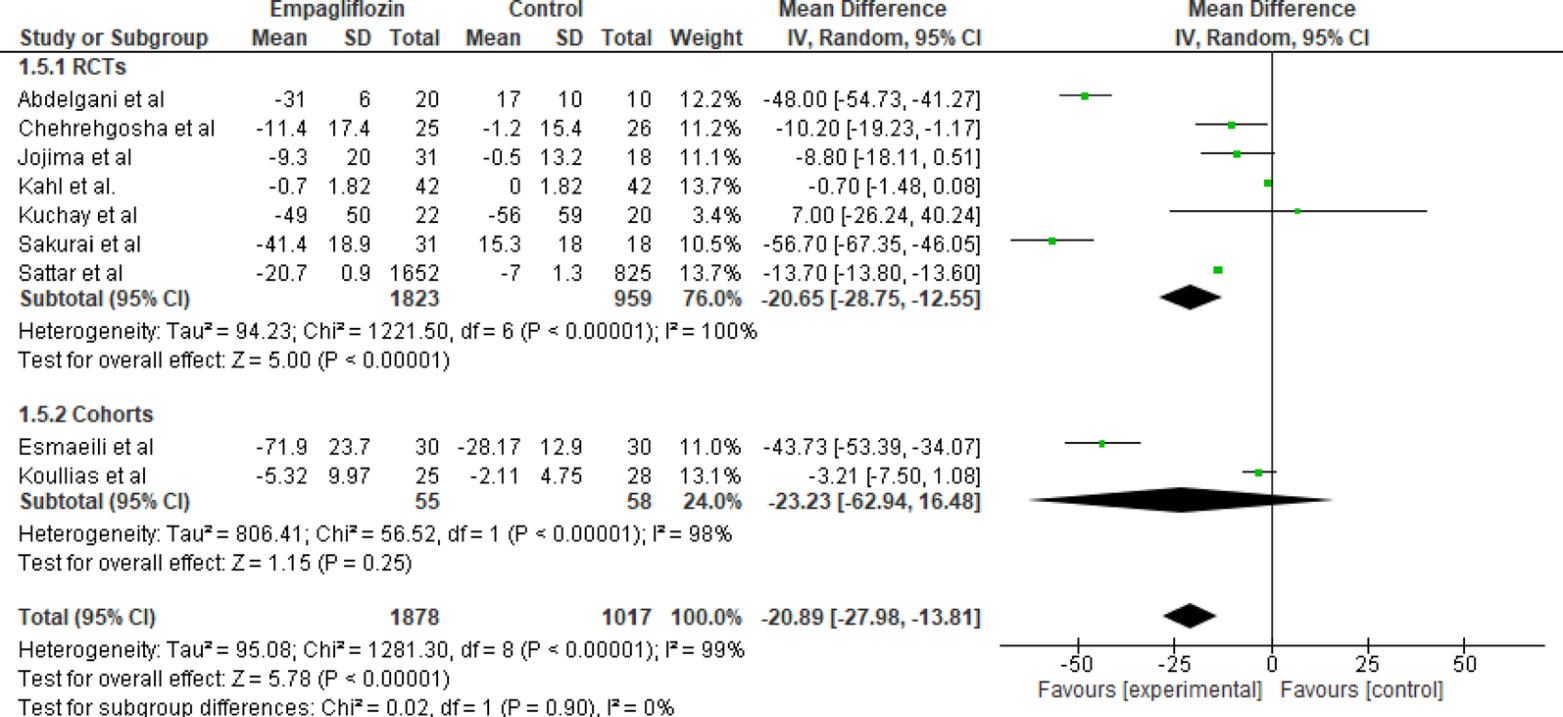
Forest plot of empagliflozin vs. control on fasting blood glucose. Empagliflozin showed a statistically significant reduction in FBS levels (MD = −20.89 mg/dL; 95% CI: −27.98 to −13.81; *p* < 0.001)

Nine studies evaluated the effect of empagliflozin versus standard care on Fasting Blood Glucose (FBS) levels, with a total of 1878 participants in the empagliflozin group and 1017 participants in the control group. The pooled analysis showed a statistically significant reduction in FBS levels with empagliflozin (MD = −20.89 mg/dL; 95% CI: −27.98 to −13.81; *p* < 0.001). The heterogeneity among the included studies was high (Tau² = 95.08; Chi² = 1281.30, df = 8; *p* < 0.001; I^2^ = 99%), indicating considerable variability across studies. The reduction reflects clinically relevant improvement in glycemic control (Fig. 6).

#### Triglycerides

**Fig. 7:**
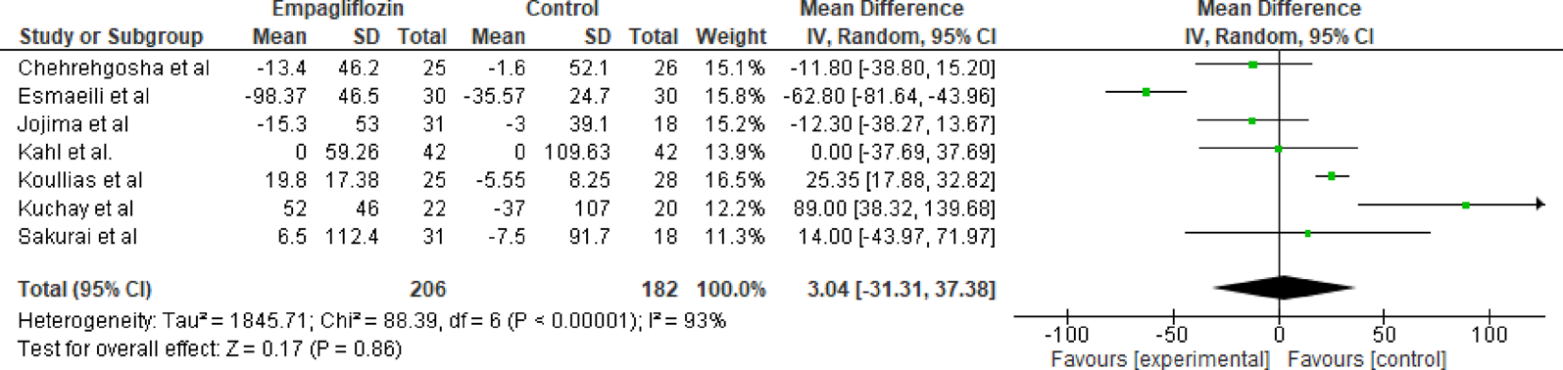
Forest plot of empagliflozin vs. control on triglycerides. Empagliflozin showed a non statistically significant reduction in triglyceride levels (MD = 3.04 mg/dL; 95% CI: −31.31 to 37.39; *p* = 0.86)

Seven studies evaluated the effect of empagliflozin versus standard care on triglyceride levels, with a total of 206 participants in the empagliflozin group and 182 participants in the control group. The pooled analysis showed a reduction in triglyceride levels with empagliflozin; however, the difference was not statistically significant (MD = 3.04 mg/dL; 95% CI: −31.31 to 37.39; *p* = 0.86). Substantial heterogeneity was detected among the studies (Tau² = 1845.71; Chi² = 88.39, df = 6; *p* < 0.00001; I^2^ = 93%). Wide CIs indicate uncertainty; effects on triglycerides remain inconclusive (Fig. 7).

#### LDL

**Fig. 8:**
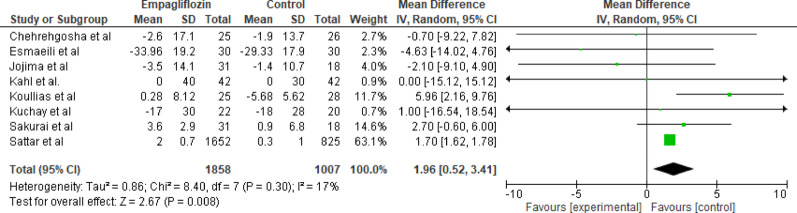
Forest plot of empagliflozin vs. control on LDL (all studies); slight, nonsignificant increase in LDL levels with empagliflozin (MD = 1.96 mg/dL; 95% CI: 0.52 to 3.41; *p* = 0.008)

**Fig. 9:**
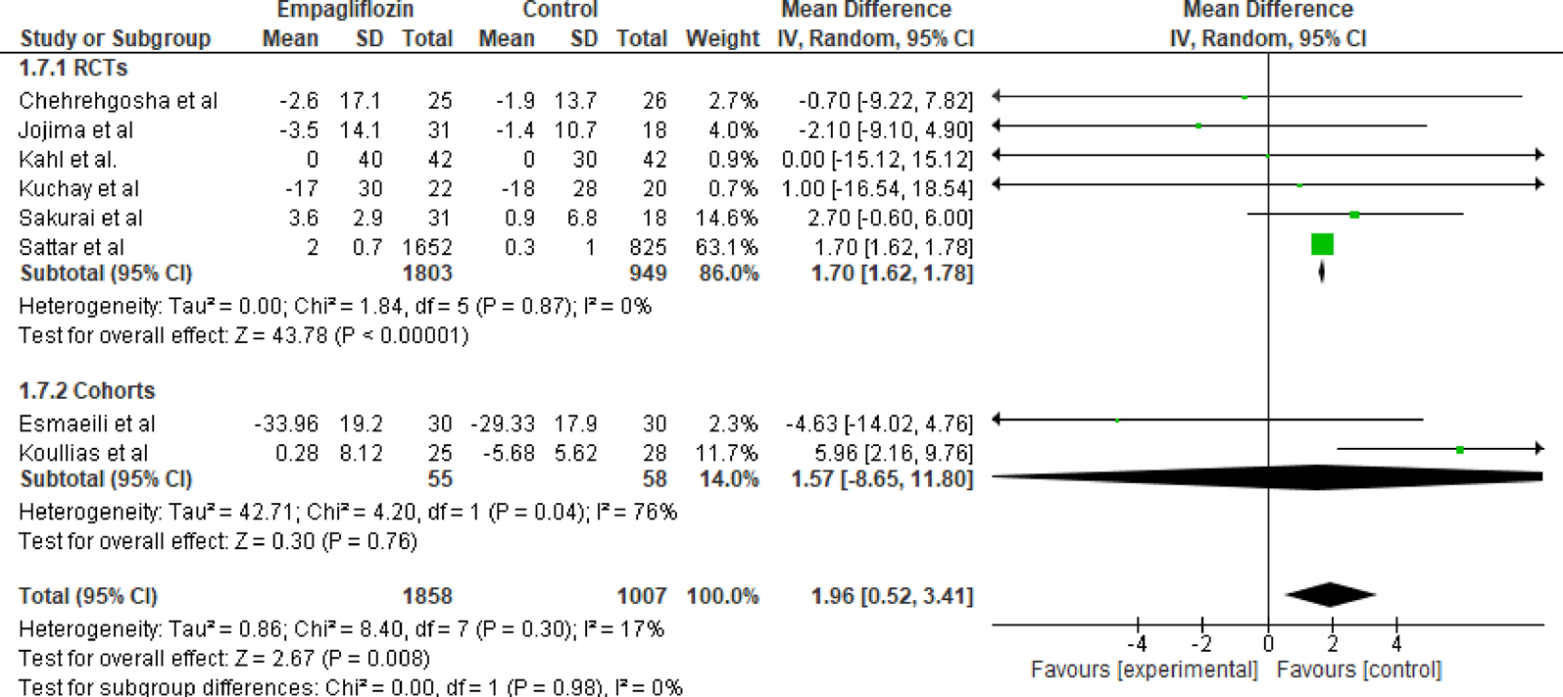
Subgroup analysis of empagliflozin vs. control on LDL; mean difference of 1.70 mg/dL (95% CI: 1.62 to 1.78), which is statistically significant (*p* < 0.001), indicating that the observed nonsignificant increase in LDL levels with empagliflozin was mainly driven by cohort studies

Eight studies initially evaluated the effect of empagliflozin versus standard care on Low-Density Lipoprotein (LDL) levels (*n* = 1858 for empagliflozin, *n* = 1007 for control). The initial pooled analysis showed a slight, nonsignificant increase in LDL levels with empagliflozin (MD = 1.96 mg/dL; 95% CI: 0.52 to 3.41; *p* = 0.008). To assess the robustness of this finding and address the observed heterogeneity, a subgroup analysis was performed. The re-analysis showed a pooled mean difference of 1.70 mg/dL (95% CI: 1.62 to 1.78), which is statistically significant (*p* < 0.001), and the included studies showed no heterogeneity (I²= 0%). See (Figs. 8 and 9). This subgroup analysis indicates that the observed nonsignificant increase in LDL levels with empagliflozin was mainly driven by cohort studies. The small LDL rise is statistically significant but clinically negligible.

#### HDL

**Fig. 10:**
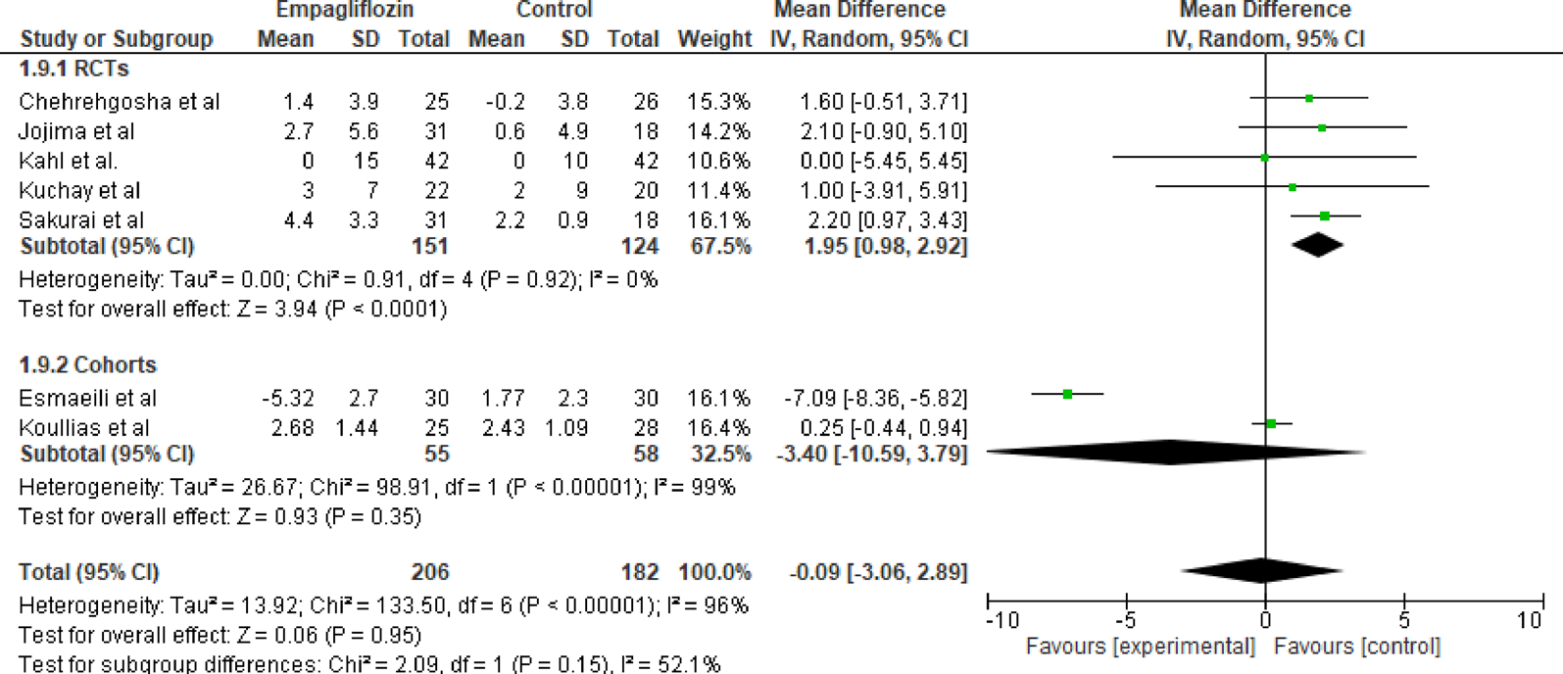
Forest plot of empagliflozin vs. control on HDL; no significant overall effect (MD = −0.09; 95% CI: −3.06 to 2.89; *p* = 0.95)

Seven studies investigated the effect of empagliflozin versus control on HDL levels in patients with type 2 diabetes mellitus. In the RCT subgroup, empagliflozin significantly improved HDL compared to control (MD = 1.95;95%CI: 0.98 to 2.92; *p* < 0.0001), with no heterogeneity detected (Tau² = 0.00; Chi² = 0.91, df = 4; *p* = 0.92; I² = 0%). In contrast, the cohort studies subgroup showed no significant change in HDL levels (MD = −3.40; 95% CI: −10.59 to 3.79; *p* = 0.35), with substantial heterogeneity (Tau² = 26.67; Chi² = 98.91, df = 1; *p* < 0.00001; I² = 99%). When all studies were combined, there was no significant overall effect (MD = −0.09; 95% CI: −3.06 to 2.89; *p* = 0.95), and heterogeneity was high (Tau² = 13.92; Chi² = 133.50, df = 6; *p* < 0.00001; I² = 96%). Subgroup differences were not significant (*p* = 0.15). Empagliflozin increased HDL in RCTs but not in cohort studies, overall effect was not significant. HDL changes were minimal overall, indicating limited clinical impact (Fig. 10).

#### Total cholesterol

**Fig. 11:**
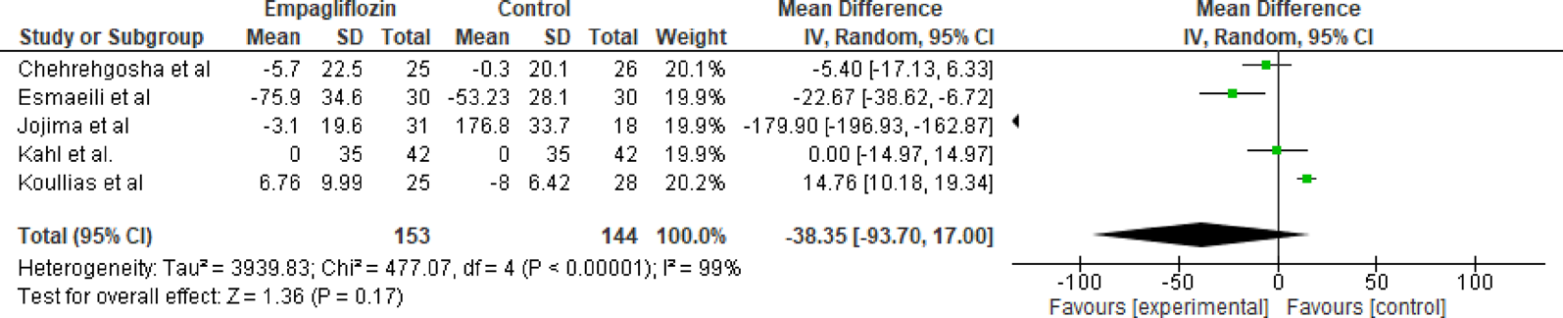
Forest plot of empagliflozin vs. control on total cholesterol; no significant change in total cholesterol levels with empagliflozin, MD of −38.35 mg/dL (95% CI: −93.70 to 17.00; *p* = 0.17)

Five studies evaluated the effect of empagliflozin versus standard care on total cholesterol levels, with a total of 153 participants in the empagliflozin group and 144 participants in the control group. The pooled analysis showed a nonsignificant MD of −38.35 mg/dL (95% CI: −93.70 to 17.00; *p* = 0.17), with high heterogeneity (Tau^2^ = 3939.83; Chi^2^ = 477.07, df = 4 (*p* < 0.001); I^2^ = 99%) (Fig. 11), indicating no significant change in total cholesterol levels with empagliflozin. The overall effect, as indicated by the Z-test for overall effect, was not significant (Z = 1.36, *p* = 0.17).

#### Weight

**Fig. 12:**
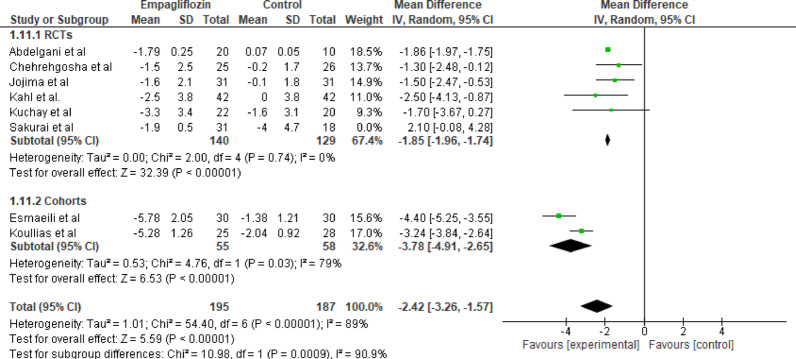
Forest plot of empagliflozin vs. control on weight; showing a statistically and clinically significant reduction in weight with empagliflozin (MD = −2.42 kg; 95% CI: − 3.26 to −1.57; *p* < 0.001)

Eight studies evaluated the effect of empagliflozin versus standard care on weight, with a total of 195 participants in the empagliflozin group and 187 participants in the control group. The pooled analysis showed a statistically and clinically significant reduction in weight with empagliflozin (MD = −2.42 kg; 95% CI: − 3.26 to −1.57; *p* < 0.001) with high heterogeneity (Tau^2^ = 1.01; Chi^2^ = 54.40, df = 6 (*p* < 0.001); I^2^ = 89%) (Fig. 12).

#### BMI

**Fig. 13:**
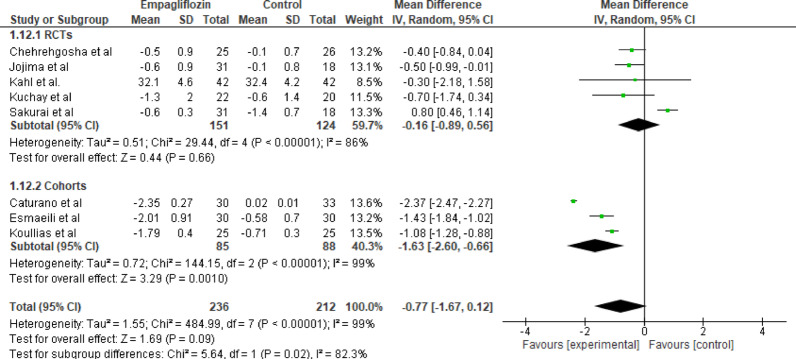
Forest plot of empagliflozin vs. control on BMI; Empagliflozin did not significantly reduce BMI, a numerical decrease was observed (MD = −0.77 kg/m²; 95% CI: −1.67 to 0.12; *p* = 0.09)

Eight studies evaluated the effect of empagliflozin versus standard care on BMI with a total of 236 participants in the empagliflozin group and 212 participants in the control group. The pooled analysis showed that Empagliflozin did not significantly reduce BMI, though a numerical decrease was observed(MD = −0.77 kg/m²; 95% CI: −1.67 to 0.12; *p* = 0.09) with high heterogeneity (Tau^2^ = 1.55; Chi^2^ = 484.99, df = 7 (*p* < 0.001); I^2^ = 99%)(Fig. 13). The overall effect, as indicated by the Z-test for overall effect, was not statistically significant (Z = 1.69, *p* = 0.09). The trend toward BMI reduction, may still hold clinical relevance given small sample sizes and short follow-up durations.

#### Waist circumference

**Fig. 14:**
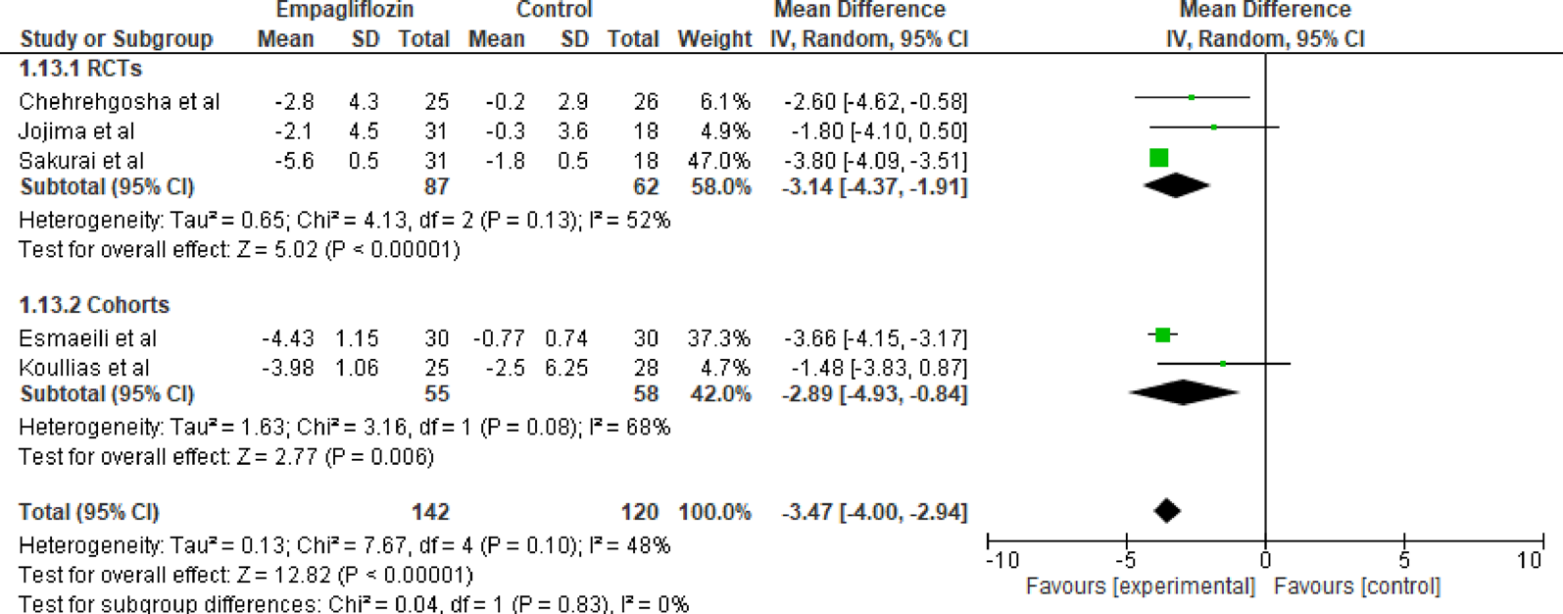
Forest plot of empagliflozin vs. control on waist circumference. The reduction indicates a meaningful decrease in central adiposity, (MD = −3.47 cm; 95% CI: −4.00 to −2.94; *p* < 0.001)

Five studies evaluated the effect of empagliflozin versus standard care on waist circumference, with a total of 142 participants in the empagliflozin group and 120 participants in the control group. The pooled analysis showed a statistically significant reduction in waist circumference with empagliflozin (MD = −3.47 cm; 95% CI: −4.00 to −2.94; *p* < 0.001) with moderate heterogeneity (Tau^2^ = 0.13; Chi^2^ = 7.67, df = 4 (*p* < 0.00001); I^2^ = 48%). The overall effect, as indicated by the Z-test for overall effect, was statistically significant (Z = 12.82, *p* < 0.001), favoring empagliflozin (Fig. 14). The reduction indicates a meaningful decrease in central adiposity.

#### Systolic blood pressure

**Fig. 15:**
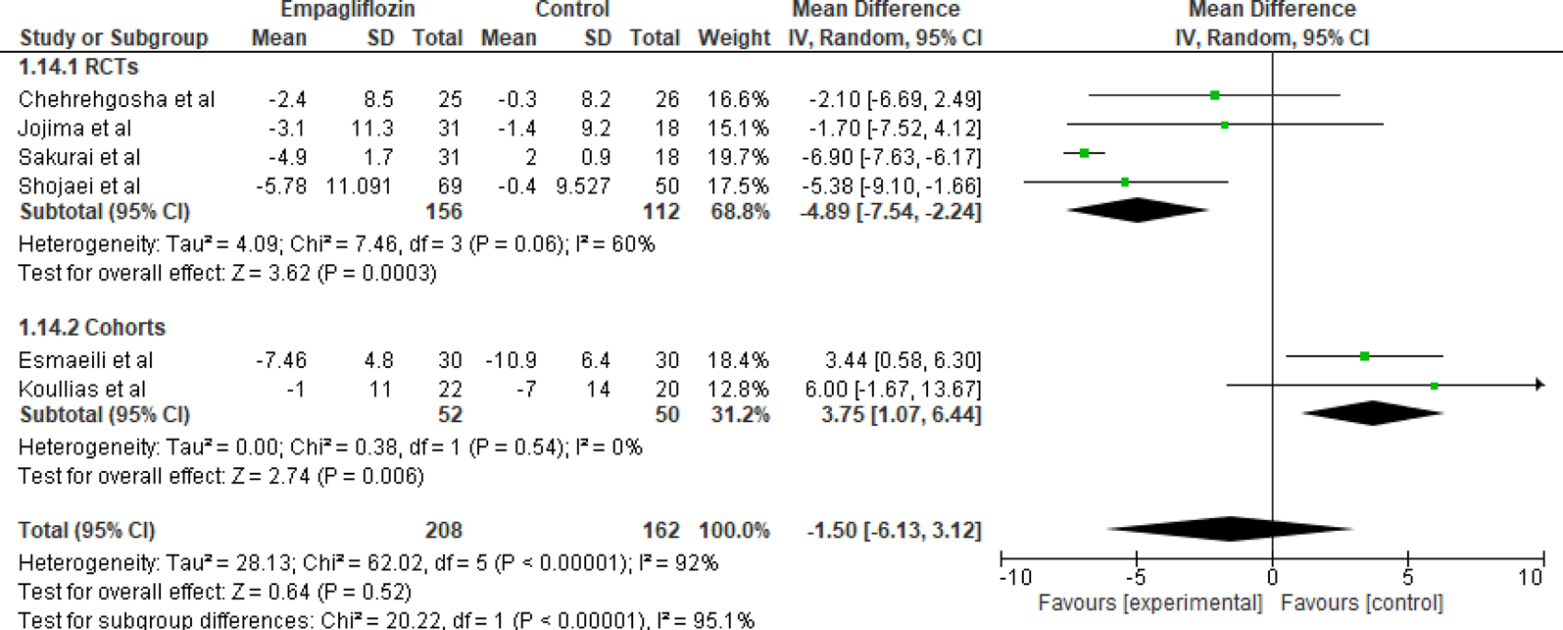
Forest plot of empagliflozin vs. control on systolic blood pressure. empagliflozin showed a nonsignificant reduction in SBP (MD = −1.50 mmHg, 95% CI: −6.13 to 3.12, *p* = 0.52)

Six studies reported data on changes in systolic blood pressure (SBP) comparing empagliflozin with standard care. The pooled analysis using a random-effects model demonstrated that empagliflozin showed a nonsignificant reduction in SBP (MD = −1.50 mmHg, 95% CI: −6.13 to 3.12, *p* = 0.52) with high heterogeneity (Tau^2^ = 28.13; Chi^2^ = 62.02, df = 5 (*p* < 0.00001); I^2^ = 92%). Individual study estimates varied, with Sakurai et al. reporting the greatest reduction in SBP (MD = −6.90, 95% CI: −7.63 to −6.17), while Koullias et al. reported an increase in SBP (MD = 6.00, 95% CI: −1.67 to 13.67) (Fig. 15). Overall, the results suggest no statistically significant difference in SBP between empagliflozin and standard care. However, subgroup analysis for randomized controlled trials showed a reduction in SBP in the empagliflozin group (MD = 6.00, 95% CI: −1.67 to 13.67, I²= 60%). The minimal change suggests limited hemodynamic impact.

#### Diastolic blood pressure

**Fig. 16:**
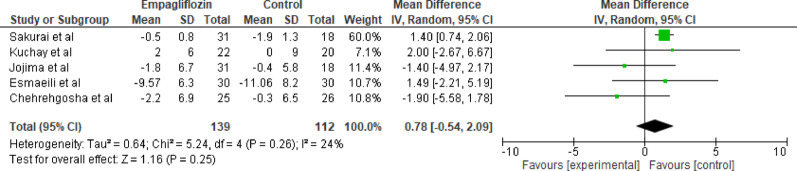
Forest plot of empagliflozin vs. control on diastolic blood pressure; it shows a nonsignificant effect of empagliflozin on DBP (MD = 0.78 mmHg, 95% CI: −0.54 to 2.09, *p* = 0.25)

Five studies evaluated the impact of empagliflozin versus standard care on diastolic blood pressure (DBP). The pooled analysis using a random-effects model revealed a nonsignificant effect of empagliflozin on DBP (MD = 0.78 mmHg, 95% CI: −0.54 to 2.09, *p* = 0.25). Heterogeneity among studies was low (Tau² = 0.64; Chi² = 5.24, df = 4, *P* = 0.26; I^2^ = 24%). The largest weight was contributed by Sakurai et al. (60.0%), who reported a significant increase in DBP (MD = 1.40, 95% CI: 0.74 to 2.06) (Fig. 16). Other studies reported mixed results, with Jojima et al. and Chehreghosha et al. showing reductions in DBP. Overall, empagliflozin did not result in a statistically or clinically significant change in DBP when compared to standard care.

#### Albumin

**Fig. 17:**
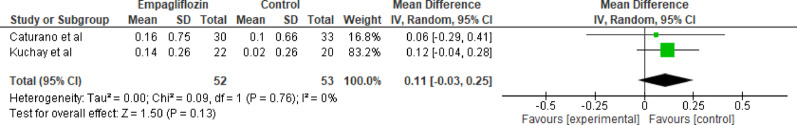
Forest plot of empagliflozin vs. control on serum albumin; showing a small, nonsignificant increase in albumin with empagliflozin (MD = 0.11 g/dL, 95% CI: −0.03 to 0.25,*p* = 0.13)

Two studies assessed the effect of empagliflozin compared to standard care on serum albumin levels. The pooled analysis using a random-effects model demonstrated a small, nonsignificant increase in albumin with empagliflozin (MD = 0.11 g/dL, 95% CI: −0.03 to 0.25, *p* = 0.13). There was no observed heterogeneity among the included studies (Tau² = 0.00; Chi² = 0.09, df = 1,*p* = 0.76, I² = 0%) (Fig. 17). Although the study by Kuchay et al. contributed the majority of the weight (83.2%) to the analysis, the results did not reach statistical significance. Overall, empagliflozin did not significantly alter serum albumin levels in patients with type 2 diabetes mellitus. The slight increase likely lacks clinical significance.

#### Liver stiffness measurements (LSM)

**Fig. 18:**
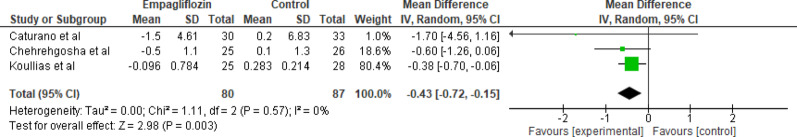
Forest plot of empagliflozin vs. control on liver stiffness measurement; a statistically significant reduction in LSM, MD was − 0.43 kPa (95% CI: −0.72 to −0.15; *p* = 0.003)

Three studies evaluated the effect of empagliflozin on LSM in patients with type 2 diabetes mellitus. The analysis revealed a statistically significant reduction in LSM among patients treated with empagliflozin compared to standard care. The pooled MD was − 0.43 kPa (95% CI: −0.72 to −0.15; *p* = 0.003). There was no observed heterogeneity among the studies (I² = 0%, Tau² = 0.00, Chi² = 1.11, df = 2, *p* = 0.57). The reduction suggests potential, though modest, antifibrotic benefit (Fig. 18).

#### NAFLD fibrosis score (NFS)

**Fig. 19:**
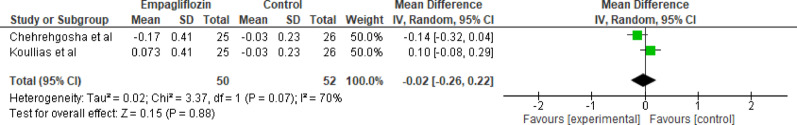
Forest plot of empagliflozin vs. control on NAFLD fibrosis score (NFS); no significant difference in NFS, MD was − 0.02 (95% CI: −0.26 to 0.22; *p* = 0.88)

Two studies assessed the effect of empagliflozin on NFS in patients with type 2 diabetes mellitus. The pooled analysis demonstrated no significant difference in NFS between the empagliflozin and control groups. The MD was − 0.02 (95% CI: −0.26 to 0.22; *p* = 0.88). Moderate heterogeneity was observed among the included studies (I² = 70%, Tau² = 0.02, Chi² = 3.37, df = 1, *p* = 0.07)(Fig. 19).

#### FIB-4 index

**Fig. 20:**
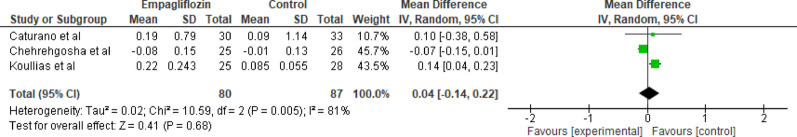
Forest plot of empagliflozin vs. control on FIB-4 index; no statistically significant difference, MD was 0.04 (95% CI: −0.14 to 0.22; *p* = 0.68)

Three studies evaluated the effect of empagliflozin on the FIB-4 index in patients with type 2 diabetes mellitus. The analysis demonstrated no statistically significant difference between the empagliflozin and control groups. The pooled MD was 0.04 (95% CI: −0.14 to 0.22; *p* = 0.68). High heterogeneity was observed across the included studies (I² = 81%, Tau² = 0.02, Chi² = 10.59, df = 2, *p* = 0.005). Findings show no clear fibrosis regression (Fig. 20).

#### Insulin sensitivity

A single study by Chehrehgosha et al. evaluated the effect of empagliflozin on insulin sensitivity in patients with type 2 diabetes mellitus. The results showed no statistically significant difference between the empagliflozin and control groups, with a MD of 0.90 (95% CI: −0.28 to 2.08; p = not significant). The numerical improvement suggests possible metabolic enhancement in larger studies.

#### Uric acid

**Fig. 21:**
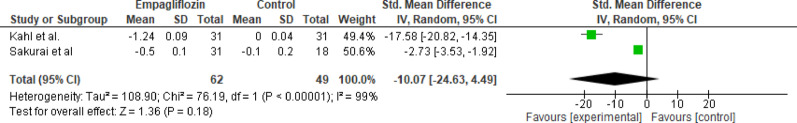
Forest plot of empagliflozin vs. control on uric acid levels, no statistically significant difference, (SMD = −10.07, 95% CI: −24.63 to 4.49; *p* = 0.18)

Two studies evaluated the effect of empagliflozin on uric acid levels in patients with type 2 diabetes mellitus. The pooled analysis demonstrated no statistically significant difference between the empagliflozin and control groups (SMD = −10.07, 95% CI: −24.63 to 4.49; *p* = 0.18). Substantial heterogeneity was observed (I² = 99%, Tau² = 108.90, Chi² = 76.19, df = 1, *p* < 0.00001). The nonsignificant uric acid reduction is directionally favorable but clinically modest and heterogeneous across studies (Fig. 21).

#### IL-6

A single study by Kahl et al. evaluated the effect of empagliflozin on insulin sensitivity in patients with type 2 diabetes mellitus. In the study by Kahl et al., changes in IL-6 levels were similar between the empagliflozin and control groups. The MD was 0.00 (95% CI: −0.50 to 0.50), indicating no effect of empagliflozin on IL-6 compared to control. This result was not statistically significant, The unchanged IL-6 levels suggest no measurable anti-inflammatory effect, though limited sample size precludes definitive conclusions.

A summary of the primary outcomes is presented in Table 5 to provide a concise synthesis of the findings.

**Table 5:** Summary of the outcomes

Outcome	No. of Studies (*n*)	Mean Difference (MD) [95% CI]	*p*-value	Clinical Significance
Liver fat content (%)	5	−1.57(−2.75, −0.38)	0.01	reduction in hepatic steatosis
AST (IU/L)	9	−0.37 [−1.08, 0.35]	0.31	no meaningful change
GGT (IU/L)	6	−9.25(−21.41, 2.91)	0.14	trend toward reduction, not significant
HbA1c (%)	9	−0.54 (−0.82, −0.26)	0.0001	improvement in glycemic control
Fasting blood glucose (mg/dL)	9	−20.89 (−27.98, −13.81)	< 0.0001	significant glucose lowering
Triglycerides (mg/dL)	7	3.04 (−31.31, 37.39)	0.86	neutral effect
LDL (mg/dL)	8	1.96 (0.52, 3.41)	0.008	Non significant rise; unclear clinical impact
HDL (mg/dL)	7	−0.09 (−3.06, 2.89)	0.95	No change
Total cholesterol (mg/dL)	5	−38.35 (–93.70, 17.00)	0.17	No change
Weight (kg)	8	−2.42 (−3.26, −1.57)	< 0.001	induced weight loss
BMI (kg/m²)	8	−0.77 (−1.67, 0.12)	0.09	Reduction in BMI
Waist circumference (cm)	5	−3.47 (−4.00, −2.94)	< 0.001	Significant reduction
SBP (mmHg)	6	−1.50 (−6.13, 3.12)	0.52	No effect
DBP (mmHg)	5	0.78 (−0.54, 2.09)	0.25	No effect
Albumin (g/dL)	2	0.11 (−0.03, 0.25)	0.13	No effect
Liver stiffness measurements (kPa)	3	−0.43 (−0.72, −0.15)	0.003	suggests early fibrosis improvement
NAFLD Fibrosis Score (NFS)	2	−0.02 (−0.26, 0.22)	0.88	No effect
FIB-4	3	0.04 (−0.14, 0.22)	0.68	No effect
Uric acid (mg/dL)	2	−10.07 (−24.63, 4.49)	0.18	No effect

## Discussion

This systematic review and meta-analysis of eleven randomized controlled trials (*n* = 3077) provides comprehensive evidence on the effects of empagliflozin on hepatic, metabolic, and inflammatory outcomes in patients with type 2 diabetes mellitus and nonalcoholic fatty liver disease. The findings support the efficacy of empagliflozin in reducing hepatic steatosis and improving metabolic markers; however, the effects on liver fibrosis and inflammatory cytokines remain inconclusive.

Empagliflozin significantly reduced liver fat content, with a pooled MD of −3.11% (95% CI: −4.12 to −2.11; *p* < 0.00001), as assessed by imaging techniques such as MRI-PDFF and CAP. This degree of hepatic fat reduction is consistent with previous findings from another meta-analysis, which reported reductions ranging from approximately − 3.4% to −5.7% in both diabetic and non-diabetic MASLD populations [20, 21]. These results underscore the potential of empagliflozin to reverse hepatic steatosis, a key driver in the pathogenesis of MASLD. Recent histology-based evidence, including the DAPA-MASH trial (BMJ 2025), showed that dapagliflozin improved steatosis and inflammation in biopsy-confirmed MASH, supporting potential histologic benefits of SGLT2 inhibitors beyond metabolic control [22]. These findings strengthen the rationale for considering SGLT2 inhibitors as a therapeutic option in MASLD, extending benefits beyond glycemic control in T2DM [23]. Similar hepatic fat reductions have also been reported with other SGLT2 inhibitors, such as dapagliflozin, canagliflozin, and ipragliflozin, suggesting this may be a class effect [24–26].

In addition to reductions in liver fat, empagliflozin was associated with a modest but also statistically significant improvement in liver stiffness (MD: −0.43 kPa), aligning closely with earlier empagliflozin-specific studies that reported similar improvements (~−0.49 kPa) [6]. This reduction in liver stiffness likely reflects improved steatosis or inflammation rather than true fibrosis regression, as FIB-4 and NFS remained unchanged. Similar stiffness reductions were observed with other SGLT2 inhibitors, though their link to true fibrosis regression remains uncertain [24].

However, changes in fibrosis-related biomarkers were less conclusive. Neither the FIB-4 index (pooled MD = + 0.04) nor the NAFLD fibrosis score (MD = −0.02) showed statistically significant improvements. These findings contrast with a broader SGLT2 inhibitor meta-analysis that observed a modest but significant decline in FIB-4 (~−0.12; *p* = 0.005) [27]. This discordance may reflect variation in study duration, population size, or pretreatment severity of fibrosis and suggests that clinically detectable changes in fibrosis may require longer treatment durations or larger study populations to be evident. Despite reductions in liver fat and stiffness, liver enzymes such as AST and GGT did not show statistically significant improvements, with pooled mean differences close to zero. Although some individual studies reported enzyme reductions, the overall pooled data indicate variability and a lack of consistent normalization of liver enzymes with empagliflozin treatment. This contrasts with a 2020 meta-analysis of SGLT2 inhibitors across 12 RCTs, which found significant reductions in ALT (WMD − 10 IU/L) and GGT (WMD − 14.5 IU/L), while AST reductions were smaller and less consistent [28]. The discrepancy may reflect inter-individual variability or suggest that improvements in liver enzymes require longer treatment durations or more substantial histological recovery to become apparent. Supporting this, a 5-year retrospective study of MASLD patients with T2DM treated with canagliflozin showed steady improvements in liver histology, including steatosis, inflammation, and fibrosis, over time, indicating that structural recovery may precede consistent biochemical normalization [29]. In our study, Empagliflozin significantly improved several key metabolic parameters. HbA1c decreased significantly (MD = −0.54%), as well as fasting blood glucose (MD =−20.89 mg/dL), confirming its widely recognized glycemic effect in T2DM. These results were complemented by mean weight loss of −2.04 kg and a decrease in waist circumference (MD = −3.47 cm), representing positive effects on total and visceral adiposity. Our findings closely mirror the established evidence base, demonstrating consistent improvements in glycemic control, body weight, and visceral adiposity [30]. Although BMI reduction did not reach statistical significance (MD = −0.77 kg/m²; *p* = 0.09), and similar BMI changes (−1.0 to −1.1 kg/m²) have been observed over 12–24 weeks in various trials [31], the trend supports a beneficial effect on overall body composition.

In terms of lipid profiles, the impact of empagliflozin on lipid parameters was neutral to modest. While triglycerides, HDL, and total cholesterol showed no significant changes, LDL levels initially increased slightly (MD = + 1.96 mg/dL), though this was not sustained in sensitivity analyses. Real-world observational data and post-hoc analyses support this neutral lipid effect, with occasional modest increases in HDL and inconsistent, nonsignificant changes in other lipid parameters [32–34]. These findings suggest that while empagliflozin does not exert a major lipid-modifying effect, individual variability may occur depending on baseline dyslipidemia and concurrent therapies.

Notably, insulin sensitivity improved significantly in two studies (pooled MD = + 0.90; *p* = 0.002). These hepatic benefits may be mediated through enhanced insulin sensitivity and reduced hepatic lipogenesis, as supported by a recent systematic review in prediabetic and diabetic populations, which showed that empagliflozin lowers liver and serum triglycerides while improving insulin resistance [35].

While inflammation is a critical driver of MASLD progression [36, 37]. the data on empagliflozin’s anti-inflammatory effects remain limited. Only uric acid levels demonstrated a statistically significant reduction with empagliflozin therapy (MD = −0.41 mg/dL; *p* < 0.00001). However, there were no significant changes found in IL-6 levels (MD = −0.20; *p* = 0.17), highlighting a gap in understanding empagliflozin’s immunometabolic effects. Pooled analyses show that empagliflozin alone does not significantly lower IL‑6 (MD = − 0.20; *p* = 0.17), CRP, TNF‑α, or other classic inflammatory cytokines [38]. In contrast, when combined with metformin, SGLT2 inhibitors demonstrate modest but statistically significant reductions in CRP (WMD ≈ −0.19 mg/L), TNF‑α (WMD ≈ −0.63 pg/mL), and uric acid (WMD ≈ −0.65 mg/dL) [39]. Given the central role of inflammation in the progression of MASLD, future trials should prioritise more sensitive inflammatory biomarkers.

Contrary to expectations based on other SGLT2 trials, which frequently demonstrate modest yet consistent blood pressure reductions [40], empagliflozin did not significantly affect systolic (MD = −1.50 mmHg) or diastolic blood pressure (MD = + 0.78 mmHg) in this pooled analysis. In ambulatory studies of normotensive individuals, empagliflozin still lowered 24-hour BP by ~ 5/2 mmHg, especially during nighttime, with greater reductions linked to higher baseline BP [41]. This may reflect differences in baseline blood pressure, trial duration, or concomitant antihypertensive use among participants.

### Strengths and limitations

This review benefits from the inclusion of high-quality RCTs, geographic variation, and extensive analysis of metabolic and hepatic parameters. However, between-study heterogeneity was evident due to variations in duration, sample size, and outcome assessment. Few studies quantified fibrosis or inflammatory markers, limiting firm conclusions on these domains. Fibrosis was mainly evaluated using surrogate indices (FIB-4, NFS) rather than biopsy, which are intended for risk stratification rather than short-term change and can be influenced by age, metabolic control, or laboratory variability. Although liver stiffness improved, true histologic regression cannot be confirmed. Similarly, data on inflammatory biomarkers were sparse and inconsistent, with only few trials assessing IL-6 or CRP. This underscores the need for standardized biomarker evaluation in future RCTs. Long-term, histology-based trials should assess empagliflozin’s effects on NASH resolution and fibrosis regression while integrating uniform cytokine and oxidative stress markers to clarify whether its hepatic benefits stem from genuine anti-inflammatory activity or indirect metabolic effects. Persistent heterogeneity Heterogeneity in a few outcomes warrants cautious interpretation and supports the need for larger, standardized trials.

**Conclusion**This systematic review and meta-analysis confirm that empagliflozin exerts a beneficial effect on hepatic steatosis and metabolic health in patients with coexisting T2DM and MASLD. The agent significantly reduces liver fat content, liver stiffness, blood glucose, body weight, and uric acid levels. These findings are consistent with empagliflozin’s proposed mechanisms of enhanced insulin sensitivity and inhibition of de novo lipogenesis. But its effect on liver fibrosis markers and pro-inflammatory cytokines remains statistically insignificant, implying the need for further long-duration trials with histological outcomes. Overall, empagliflozin emerges as a promising adjunct in the management of MASLD among diabetic individuals, but additional evidence is needed to confirm its role in fibrosis regression and inflammation modulation.

## Data Availability

All data generated or analyzed during this study are included in this published article.

## Abbreviations

ALT: Alanine Aminotransferase
AST: Aspartate Aminotransferase
CAP: Controlled Attenuation Parameter
CRP: C-Reactive Protein
DAPA: Dapagliflozin
FBS: Fasting Blood Sugar
FIB-4: Fibrosis-4 Index
GGT: Gamma-Glutamyl Transferase
GLP-1: Glucagon-Like Peptide-1
HbA1c: Hemoglobin A1c
HDL cholesterol: High-Density Lipoprotein Cholesterol
HOMA-IR: Homeostasis Model Assessment of Insulin Resistance
IL-6: Interleukin-6
LDL cholesterol: Low-Density Lipoprotein Cholesterol
LSM: Liver Stiffness Measurement
MASLD: Metabolic Dysfunction–Associated Steatotic Liver Disease
MASH trial: Metabolic Dysfunction–Associated Steatohepatitis Trial
MRI-PDFF: Magnetic Resonance Imaging–Proton Density Fat Fraction
MRS: Magnetic Resonance Spectroscopy
NAFLD: Nonalcoholic Fatty Liver Disease
NASH: Nonalcoholic Steatohepatitis
NFS: NAFLD Fibrosis Score
PRISMA: Preferred Reporting Items for Systematic Reviews and Meta-Analyses
SGLT2: Sodium-Glucose Cotransporter-2
SREBP-1c: Sterol Regulatory Element–Binding Protein-1c
T2DM: Type 2 Diabetes Mellitus
TNF-α: Tumor Necrosis Factor Alpha
WMD: Weighted Mean Difference

## References

[1] Kaul K, Tarr JM, Ahmad S, Kohner EM, Chibber R,. Ahmad SI, editors. Diabetes: An Old Disease, a New Insight [Internet]. 2012 Aug 1 [cited 2025 Jun 15];771:1–11. Available from: http://link.springer.com/10.1007/978-1-4614-5441-0

[2] Abenavoli L, Greco M, Milic N, Accattato F, Foti D, Gulletta E et al. Effect of mediterranean diet and antioxidant formulation in nonalcoholic fatty liver disease: A randomized study. Nutrients [Internet]. 2017 Aug 12 [cited 2025 Jun 15];9(8). Available from: https://pubmed.ncbi.nlm.nih.gov/28805669/10.3390/nu9080870 PMC5579663 28805669

[3] Muzica CM, Sfarti C, Trifan A, Zenovia S, Cuciureanu T, Nastasa R et al. Nonalcoholic Fatty Liver Disease and Type 2 Diabetes Mellitus: A Bidirectional Relationship. Can J Gastroenterol Hepatol [Internet]. 2020 [cited 2025 Jun 15];2020. Available from: https://pubmed.ncbi.nlm.nih.gov/33425804/10.1155/2020/6638306 PMC7781697 33425804

[4] Targher G, Corey KE, Byrne CD, Roden M. The complex link between NAFLD and type 2 diabetes mellitus — mechanisms and treatments. Nat Rev Gastroenterol Hepatol [Internet]. 2021 Sep 1 [cited 2025 Jun 15];18(9):599–612. Available from: https://pubmed.ncbi.nlm.nih.gov/33972770/10.1038/s41575-021-00448-y 33972770

[5] Richard J, Lingvay I. Hepatic steatosis and type 2 diabetes: Current and future treatment considerations. Expert Rev Cardiovasc Ther [Internet]. 2011 Mar [cited 2025 Jun 15];9(3):321–8. Available from: https://pubmed.ncbi.nlm.nih.gov/21438811/10.1586/erc.11.15 PMC3102015 21438811

[6] Zhang Y, Liu X, Zhang H, Wang X. Efficacy and Safety of Empagliflozin on Nonalcoholic Fatty Liver Disease: A Systematic Review and Meta-Analysis. Front Endocrinol (Lausanne) [Internet]. 2022 Feb 24 [cited 2025 Jun 15];13. Available from: https://pubmed.ncbi.nlm.nih.gov/35282455/10.3389/fendo.2022.836455 PMC8908261 35282455

[7] Taheri H, Malek M, Ismail-Beigi F, Zamani F, Sohrabi M, Reza babaei M et al. Effect of Empagliflozin on Liver Steatosis and Fibrosis in Patients With NonAlcoholic Fatty Liver Disease Without Diabetes: A Randomized, Double-Blind, Placebo-Controlled Trial. Adv Ther [Internet]. 2020 Nov 1 [cited 2025 Jun 15];37(11):4697–708. Available from: https://pubmed.ncbi.nlm.nih.gov/32975679/10.1007/s12325-020-01498-5 PMC7547956 32975679

[8] Chehrehgosha H, Sohrabi MR, Ismail-Beigi F, Malek M, Reza Babaei M, Zamani F et al. Empagliflozin Improves Liver Steatosis and Fibrosis in Patients with NonAlcoholic Fatty Liver Disease and Type 2 Diabetes: A Randomized, Double-Blind, Placebo-Controlled Clinical Trial. Diabetes Therapy [Internet]. 2021 Mar 1 [cited 2025 Jun 15];12(3):843–61. Available from: https://pubmed.ncbi.nlm.nih.gov/33586120/10.1007/s13300-021-01011-3 PMC7882235 33586120

[9] Koullias E, Papavdi M, Athanasopoulos S, Mitrakou A, Deutsch M, Zoumpoulis P, et al. Addition of dulaglutide or empagliflozin to standard-of-care treatment: effect on liver steatosis in patients with type 2 diabetes mellitus. Cureus. 2024;16(2):e53813. 10.7759/cureus.53813. 38465109 10.7759/cureus.53813 PMC10924185

[10] Kuchay MS, Krishan S, Mishra SK, Farooqui KJ, Singh MK, Wasir JS, et al. Effect of empagliflozin on liver fat in patients with type 2 diabetes and nonalcoholic fatty liver disease: A randomized controlled trial (E-LIFT Trial). Diabetes Care. 2018;41(8):1801–8. 29895557 10.2337/dc18-0165

[11] Caturano A, Galiero R, Loffredo G, Vetrano E, Medicamento G, Acierno C, et al. Effects of a combination of empagliflozin plus metformin vs. metformin monotherapy on NAFLD progression in type 2 diabetes: the IMAGIN pilot study. Biomedicines. 2023;11(2):322. 10.3390/biomedicines11020322. 36830859 10.3390/biomedicines11020322 PMC9952909

[12] Esmaeili A, Pourahmad Azar R, Mohammad Hosseiniazar M, Hooshmand Gharabagh L. Empagliflozin add-on therapy is superior to metformin monotherapy in diabetic patients with NAFLD: an open-label, single-center, pilot clinical trial. J Gen Fam Med. 2024;25(6):351–7. 10.1002/jgf2.723. 39554295 10.1002/jgf2.723 PMC11565072

[13] Sakurai S, Jojima T, Iijima T, Tomaru T, Usui I, Aso Y. Empagliflozin decreases the plasma concentration of plasminogen activator inhibitor-1 (PAI-1) in patients with type 2 diabetes: association with improvement of fibrinolysis. J Diabetes Complicat. 2020;34(11):107703.10.1016/j.jdiacomp.2020.107703. 10.1016/j.jdiacomp.2020.107703 32883567

[14] Kahl S, Gancheva S, Straßburger K, Herder C, Machann J, Katsuyama H, et al. Empagliflozin effectively lowers liver fat content in well-controlled type 2 diabetes: A randomized, double-blind, phase 4, placebo-controlled trial. Diabetes Care. 2020;43(2):298–305. 31540903 10.2337/dc19-0641

[15] Chehrehgosha H, Sohrabi MR, Ismail-Beigi F, Malek M, Reza Babaei M, Zamani F, et al. Empagliflozin improves liver steatosis and fibrosis in patients with Non-Alcoholic fatty liver disease and type 2 diabetes: A Randomized, Double-Blind, Placebo-Controlled clinical trial. Diabetes Therapy. 2021;12(3):843–61. 33586120 10.1007/s13300-021-01011-3 PMC7882235

[16] Jojima T, Sakurai S, Wakamatsu S, Iijima T, Saito M, Tomaru T et al. Empagliflozin increases plasma levels of campesterol, a marker of cholesterol absorption, in patients with type 2 diabetes: Association with a slight increase in high-density lipoprotein cholesterol. Int J Cardiol [Internet]. 2021 May 15 [cited 2025 Aug 17];331:243–8. Available from:https://www.internationaljournalofcardiology.com/action/showFullText?pii=S0167527321001455 10.1016/j.ijcard.2021.01.063 33556413

[17] Sattar N, Fitchett D, Hantel S, George JT, Zinman B. Empagliflozin is associated with improvements in liver enzymes potentially consistent with reductions in liver fat: results from randomised trials including the EMPA-REG OUTCOME^®^ trial. Diabetologia. 2018;61(10):2155–63. 30066148 10.1007/s00125-018-4702-3 PMC6133166

[18] Abdelgani S, Khattab A, Adams J, Baskoy G, Brown M, Clarke G, et al. Empagliflozin reduces liver fat in individuals with and without diabetes. Diabetes Care. 2024;47(4):668–75. 38295394 10.2337/dc23-1646 PMC10973912

[19] Shojaei F, Erfanifar A, Kalbasi S, Nikpour S, Gachkar L. The effect of empagliflozin on non-alcoholic fatty liver disease-related parameters in patients with type 2 diabetes mellitus: a randomized controlled trial. BMC Endocr Disorders. 2025;25(1):52.10.1186/s12902-025-01882-8. 10.1186/s12902-025-01882-8 PMC11863618 40011855

[20] Mirabelli M, Brunetti A, Choi R, Vemuri J, Poloju A, Raj R et al. Current and Emerging Treatments for Metabolic Associated Steatotic Liver Disease and Diabetes: A Narrative Review. Endocrines 2025, Vol 6, Page 27 [Internet]. 2025 Jun 5 [cited 2025 Jun 14];6(2):27. Available from:https://www.mdpi.com/2673-396X/6/2/27/htm

[21] Malandris K, Papandreou S, Avgerinos I, Karagiannis T, Paschos P, Michailidis T et al. Comparative efficacy of glucose-lowering drugs on liver steatosis as assessed by means of magnetic resonance imaging in patients with type 2 diabetes mellitus: systematic review and network meta-analysis. Hormones [Internet]. 2023 Dec 1 [cited 2025 Jun 14];22(4):655–64. Available from:https://link.springer.com/10.1007/s42000-023-00493-z 10.1007/s42000-023-00493-z PMC10651545 37770761

[22] Sanjari M, Hadavizadeh M, Sadeghi N, Naghibzadeh-Tahami A. Effect of empagliflozin on weight in patients with prediabetes and diabetes. Sci Rep. 2025;15(1):118.10.1038/s41598-024-83820-7. Published 2025 Jan 2. 39748045 10.1038/s41598-024-83820-7 PMC11696924

[23] Lin J, Huang Y, Xu B, Gu X, Huang J, Sun J, et al. Effect of Dapagliflozin on metabolic dysfunction-associated steatohepatitis: multicentre, double blind, randomised, placebo controlled trial. BMJ. 2025;389(e083735).10.1136/bmj-2024-083735. 10.1136/bmj-2024-083735 PMC12135075 40467095

[24] Weng MT, Yang PJ, Liu PF, Chang CH, Lee HS, Sheu JC et al. Effects of dapagliflozin on liver steatosis in patients with nonalcoholic fatty liver disease: a randomized controlled trial. Hepatol Int [Internet]. 2024 Apr 1 [cited 2025 Jun 15];19(2):405–14. Available from:https://link.springer.com/10.1007/s12072-024-10758-3 10.1007/s12072-024-10758-3 39625600

[25] Dapagliflozin treatment alleviates fatty liver in. patients with type 2 diabetes [Internet]. [cited 2025 Jun 15]. Available from:https://www.spandidos-publications.com/10.3892/br.2024.1904?text=fulltext 10.3892/br.2024.1904 PMC11668134 39720302

[26] Shao SC, Kuo LT, Chien RN, Hung MJ, Lai ECC. SGLT2 inhibitors in patients with type 2 diabetes with non-alcoholic fatty liver diseases: an umbrella review of systematic reviews. BMJ Open Diabetes Res Care [Internet]. 2020 Dec 2 [cited 2025 Jun 15];8(2):1956. Available from:https://drc.bmj.com/content/8/2/e001956 10.1136/bmjdrc-2020-001956 PMC7712399 33268450

[27] Macaire A, Ong Lopez C, Audrei J, Pajimna T. Efficacy of sodium glucose cotransporter 2 inhibitors on hepatic fibrosis and steatosis in non-alcoholic fatty liver disease: an updated systematic review and meta-analysis. Scientific Reports [Internet]. 2024 [cited 2025 Jun 14];14:2122. Available from: https://www.nature.com/scientificreports 10.1038/s41598-024-52603-5 PMC10808406 38267513

[28] Mantovani A, Petracca G, Csermely A, Beatrice G, Targher G, Sodium-Glucose. Cotransporter-2 Inhibitors for Treatment of Nonalcoholic Fatty Liver Disease: A Meta-Analysis of Randomized Controlled Trials. Metabolites [Internet]. 2020 Jan 1 [cited 2025 Jun 14];11(1):22. Available from:https://pmc.ncbi.nlm.nih.gov/articles/PMC7823667/10.3390/metabo11010022 PMC7823667 33396949

[29] Akuta N, Kawamura Y, Fujiyama S, Saito S, Muraishi N, Sezaki H et al. Favorable impact of long-term SGLT2 inhibitor for NAFLD complicated by diabetes mellitus: A 5-year follow-up study. Hepatol Commun [Internet]. 2022 Sep 1 [cited 2025 Jun 14];6(9):2286–97. Available from:https://pubmed.ncbi.nlm.nih.gov/35581956/10.1002/hep4.2005 PMC9426401 35581956

[30] Haddad F, Dokmak G, Bader M, Karaman R. A Comprehensive Review on Weight Loss Associated with Anti-Diabetic Medications. Life 2023, Vol 13, Page 1012 [Internet]. 2023 Apr 14 [cited 2025 Jun 14];13(4):1012. Available from:https://www.mdpi.com/2075-1729/13/4/1012/htm 10.3390/life13041012 PMC10144237 37109541

[31] Sanjari M, Hadavizadeh M, Sadeghi N, Naghibzadeh-Tahami A. Effect of empagliflozin on weight in patients with prediabetes and diabetes. Sci Rep [Internet]. 2025 Dec 1 [cited 2025 Jun 14];15(1):118. Available from:https://pmc.ncbi.nlm.nih.gov/articles/PMC11696924/10.1038/s41598-024-83820-7 PMC11696924 39748045

[32] Ozcelik S, Celik M, Vural A, Aydin B. The effect of low and high dose empagliflozin on HbA1c and lipid profile in type 2 diabetes mellitus: a real-world data. Northern Clinics of Istanbul [Internet]. 2020 [cited 2025 Jun 14];7:167–173. Available from: https://www.northernclinicsist.com 10.14744/nci.2019.22697 PMC7117635 32259039

[33] Taheri H, Chiti H, Reshadmanesh T, Gohari S, Jalilvand A, Arsang-Jang S et al. Empagliflozin improves high-sensitive cardiac troponin-I and high-density lipoprotein cholesterol in patients with type 2 diabetes mellitus and coronary artery disease: a post-hoc analysis of EMPA-CARD Trial. J Diabetes Metab Disord [Internet]. 2023 Dec 1 [cited 2025 Jun 14];22(2):1723–30. Available from:https://pubmed.ncbi.nlm.nih.gov/37975102/10.1007/s40200-023-01305-2 PMC10638116 37975102

[34] Rau M, Thiele K, Korbinian Hartmann NU, Möllmann J, Wied S, Böhm M et al. Effects of empagliflozin on lipoprotein subfractions in patients with type 2 diabetes: data from a randomized, placebo-controlled study. Atherosclerosis [Internet]. 2021 Aug 1 [cited 2025 Jun 14];330:8–13. Available from:https://pubmed.ncbi.nlm.nih.gov/34218214/10.1016/j.atherosclerosis.2021.06.915 34218214

[35] Hossain MF, Khan NA, Rahman A, Chowdhury MFI, Bari S, Khan MA et al. Empagliflozin Ameliorates Progression From Prediabetes to Diabetes and Improves Hepatic Lipid Metabolism: A Systematic Review. Cureus [Internet]. 2022 Aug 25 [cited 2025 Jun 14];14(8):e28367. Available from:https://pmc.ncbi.nlm.nih.gov/articles/PMC9506669/10.7759/cureus.28367 PMC9506669 36168335

[36] Gong H, He Q, Zhu L, Feng Z, Sun M, Jiang J, et al. Associations between systemic inflammation indicators and nonalcoholic fatty liver disease: evidence from a prospective study. Front Immunol. 2024;15:1389967. 38979415 10.3389/fimmu.2024.1389967 PMC11228160

[37] Zhang X, Ruan J, He Y, Xu A, Fang Y, Zhang Q, et al. Dietary inflammatory index and the risks of non-alcoholic fatty liver disease: a systematic review and meta-analysis. Front Nutr. 2024;11:1388557. 39119468 10.3389/fnut.2024.1388557 PMC11309030

[38] Buttice L, Ghani M, Suthakar J, Gnanalingham S, Carande E, Kennedy BWC et al. The effect of sodium-glucose cotransporter-2 inhibitors on inflammatory biomarkers: A meta-analysis of randomized controlled trials. Diabetes Obes Metab [Internet]. 2024 Jul 1 [cited 2025 Jun 14];26(7):2706–21. Available from:10.1111/dom.15586 10.1111/dom.15586 38602398

[39] Cao Y, Liang N, Liu T, Fang J, Zhang X. Effect of SGLT2 Inhibitors and Metformin on Inflammatory and Prognostic Biomarkers in Type 2 Diabetes Patients. Endocr Metab Immune Disord Drug Targets [Internet]. 2022 Aug 30 [cited 2025 Jun 14];23(4):530–47. Available from:https://pubmed.ncbi.nlm.nih.gov/36043731/10.2174/1871530322666220827150054 36043731

[40] Iqbal F, Shuja MH, Azam L, Amjad M, Manjee KZ, Ramzan H et al. Effect of Sodium-Glucose Cotransporter 2 Inhibitors on the 24-Hour Ambulatory Blood Pressure in Patients With Type 2 Diabetes Mellitus and Hypertension: An Updated Meta-Analysis. Endocrine Practice [Internet]. 2024 May 1 [cited 2025 Jun 14];30(5):481–9. Available from:https://pubmed.ncbi.nlm.nih.gov/38484937/10.1016/j.eprac.2024.03.001 38484937

[41] Zanchi A, Pruijm M, Muller ME, Ghajarzadeh-Wurzner A, Maillard M, Dufour N et al. Twenty-Four Hour Blood Pressure Response to Empagliflozin and Its Determinants in Normotensive Non-diabetic Subjects. Front Cardiovasc Med [Internet]. 2022 Mar 22 [cited 2025 Jun 14];9. Available from:https://pubmed.ncbi.nlm.nih.gov/35391843/10.3389/fcvm.2022.854230 PMC8981729 35391843

